# Integrated network pharmacology, molecular docking, and experimental validation elucidate the anti-inflammatory and antioxidant mechanisms of apigenin in LPS-induced acute lung injury

**DOI:** 10.1039/d6ra05523k

**Published:** 2026-07-23

**Authors:** Xianglong Kong, Yan Liu, Zhigou Zhou, Liangdong Zhu, Xia Ai, Jiefu Tang, Jianjin Guo, Peng Tian, Xia Chen

**Affiliations:** a The First Hospital of Changsha Changsha 410000 Hunan Province China; b The First Affiliated Hospital of Hunan University of Medicine Huaihua 418000 Hunan Province China 492880114@qq.com 184895725@qq.com; c State Key Laboratory of Natural Product Chemistry, College of Chemistry and Chemical Engineering, Lanzhou University Lanzhou 730000 China gjj960326@163.com

## Abstract

Acute lung injury (ALI) and its severe form, acute respiratory distress syndrome (ARDS), are associated with high clinical mortality and lack effective therapeutic agents. The natural flavonoid apigenin possesses well-defined anti-inflammatory and antioxidant activities; however, its protective mechanism in ALI remains to be systematically elucidated. In this study, we established LPS-induced mouse models of ALI and BEAS-2B human bronchial epithelial cell injury models, combined with network pharmacology, molecular docking, and 100 ns molecular dynamics simulations, and employed the ferroptosis inhibitor Fer-1 and inducer Erastin for mechanistic validation, to comprehensively evaluate the protective effects of apigenin. Our results demonstrated that apigenin dose-dependently alleviated pulmonary histopathological damage, reduced inflammatory cell infiltration, myeloperoxidase activity, and the levels of pro-inflammatory cytokines IL-6, IL-1β, and TNF-α. Concurrently, apigenin inhibited the phosphorylation of NF-κB and JAK2-STAT3 pathways, upregulated the expression of GPX4 and SLC7A11, decreased Fe^2+^ and malondialdehyde levels, and attenuated lipid peroxidation. These effects were similar to those of Fer-1 and were partially reversed by Erastin. Network pharmacology and molecular simulations revealed that apigenin stably binds to core targets including MMP9, EGFR, and ESR1, and KEGG enrichment analysis significantly pointed to the NF-κB and JAK-STAT pathways. Collectively, apigenin effectively alleviates LPS-induced ALI through coordinated regulation of the NF-κB/JAK2-STAT3 pathway and inhibition of inflammatory responses, ferroptosis, and oxidative stress, thus providing a novel theoretical basis and a candidate therapeutic strategy for the treatment of ALI with this flavonoid.

## Abstract 2

Apigenin alleviates LPS-induced ALI *via* NF-κB/JAK2-STAT3 and ferroptosis inhibition through GPX4/SLC7A11, validated by network pharmacology, MD, and experiments.

## Graphical abstract

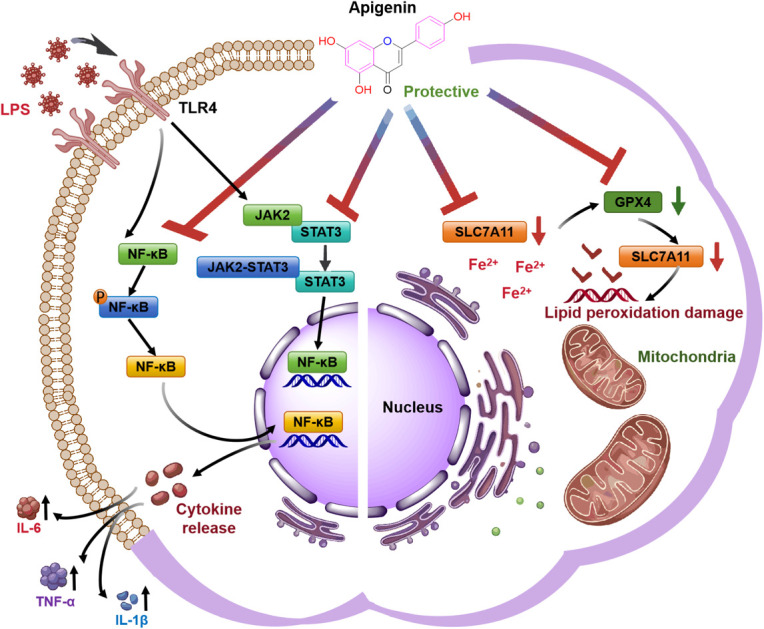

## 1. Introduction

Acute lung injury (ALI) and its more severe counterpart, acute respiratory distress syndrome (ARDS), represent life-threatening pulmonary disorders frequently triggered by severe infection, trauma, or shock. These conditions are hallmarked by disruption of the alveolar-capillary barrier, pulmonary edema, and refractory hypoxemia, contributing to mortality rates of 30–40%.^1–4^ To date, clinical management remains largely supportive, with mechanical ventilation and infection control as the mainstays, while effective targeted pharmacotherapies are still lacking.^5^ Accordingly, there is an urgent need to better understand the underlying mechanisms of ALI and to identify safe, efficacious therapeutic candidates.

Dysregulated inflammation is widely recognized as a key driver in the pathogenesis of ALI.^6,7^ Upon exposure to lipopolysaccharide (LPS), a major component of Gram-negative bacterial outer membranes, toll-like receptor 4 (TLR4) is activated, triggering a cascade of downstream inflammatory signals. Among these, the transcription factors nuclear factor kappa-B (NF-κB) and signal transducer and activator of transcription 3 (STAT3) serve as critical nodes that amplify pro-inflammatory responses.^8,9^ NF-κB activation promotes the transcriptional upregulation of pro-inflammatory cytokines such as IL-6, IL-1β, and TNF-α, while the Janus kinase 2 (JAK2)-STAT3 axis further exacerbates the inflammatory environment, together contributing to progressive lung tissue damage.^10,11^ Given their synergistic effects, simultaneous inhibition of the NF-κB and JAK2-STAT3 pathways has been proposed as a promising therapeutic approach for ALI.^12^

In recent years, ferroptosis—a distinct form of regulated cell death—has been increasingly implicated in the pathogenesis of ALI.^13,14^ This process is driven by iron-dependent lipid peroxide accumulation, with glutathione peroxidase 4 (GPX4) serving as a critical protective factor that reduces toxic lipid peroxides to non-toxic lipid alcohols, thereby preserving membrane integrity.^15–17^ Solute carrier family 7 member 11 (SLC7A11) is another key regulator involved in this process. Experimental evidence indicates that LPS stimulation suppresses GPX4 expression, elevates Fe^2+^ levels, and promotes the accumulation of lipid peroxidation byproducts such as malondialdehyde (MDA) and 4-hydroxynonenal (4-HNE) in lung tissue, pointing to ferroptosis as a key pathological event in ALI.^18^ Importantly, inflammation and ferroptosis appear to mutually reinforce one another: activation of NF-κB drives the production of inflammatory cytokines, which in turn exacerbate oxidative stress and ferroptosis; conversely, lipid peroxides generated during ferroptosis can further amplify inflammatory signaling, forming a self-sustaining vicious cycle.^19,20^ Therefore, therapeutic strategies that concurrently target both inflammatory pathways and ferroptosis may yield synergistic protective effects in the lung.

Apigenin is a naturally occurring flavonoid abundantly present in various plants, including celery, chamomile, and parsley, and exhibits diverse pharmacological properties, including anti-inflammatory, antioxidant, and anti-tumor activities.^21–24^ Previous studies have demonstrated that apigenin protects against paraquat-induced lung injury;^25^ however, its effects on the NF-κB pathway, JAK2-STAT3 signaling, and ferroptosis in the context of LPS-induced ALI have not been systematically investigated. Therefore, this study aims to systematically elucidate the mechanism by which apigenin alleviates LPS-induced ALI through integrated *in vivo* and *in vitro* experiments, combined with network pharmacology, molecular docking, and molecular dynamics simulations. Specifically, we focus on clarifying its regulatory effects on the NF-κB/JAK2-STAT3 pathway and its impact on ferroptosis, thereby providing a novel theoretical foundation for the therapeutic application of apigenin in ALI. In addition, the integration of network pharmacology with molecular docking and 100 ns molecular dynamics simulations offers chemical-level insights into the molecular recognition and binding stability between apigenin and its core targets, providing structure–activity relationship clues that complement the pharmacological data.

## 2. Materials and methods

### 2.1. Chemicals and reagents

Apigenin (HPLC ≥98%) and the ferroptosis inhibitor Ferrostatin-1 (Fer-1) were purchased from Shanghai Yuanye Biotechnology Co., Ltd (Shanghai, China). Lipopolysaccharide (LPS) was obtained from Sigma-Aldrich (USA), and the ferroptosis inducer Erastin was purchased from MedChemExpress (MCE, USA). The Cell Counting Kit-8 (CCK-8) was obtained from Biolite Biotechnology Co., Ltd (Guangzhou, China), and the lactate dehydrogenase (LDH) cytotoxicity assay kit was purchased from Wanlei Biotechnology Co., Ltd (Shenyang, China). Enzyme-linked immunosorbent assay (ELISA) kits for IL-6, IL-1β, and TNF-α, as well as antibodies against GPX4, SLC7A11, and 4-HNE, were obtained from Arigo Biolaboratories (Taiwan, China). Assay kits for superoxide dismutase (SOD), glutathione (GSH), MDA, and myeloperoxidase (MPO) were purchased from Nanjing Jiancheng Bioengineering Institute (Nanjing, China). The ferrous ion (Fe^2+^) detection kit was obtained from Jiangsu Aidisheng Biotechnology Co., Ltd (Jiangsu, China). Antibodies against NF-κB pathway components (p65, p-p65, IκBα, p-IκBα) and JAK2-STAT3 pathway components (JAK2, p-JAK2, STAT3, p-STAT3) were purchased from Cell Signaling Technology (USA). β-Actin antibody and horseradish peroxidase (HRP)-conjugated secondary antibodies were obtained from Wuhan Sanying Biotechnology Co., Ltd (Wuhan, China). The fluorescent dye FerroOrange was purchased from Dojindo (Japan), while MitoSOX Red and C11-BODIPY were obtained from Thermo Fisher Scientific (USA). Other routine reagents, including PBS, penicillin-streptomycin solution, trypsin-EDTA, RIPA lysis buffer, BCA protein assay kit, SDS-PAGE gel preparation kit, and ECL chemiluminescence reagent, were purchased from Beyotime Biotechnology (Shanghai, China).

### 2.2. Animal experimental design and treatment

All animal procedures were approved by the Medical Ethics Committee of Hunan University of Medicine (Approval No.: 202603076). Male C57BL/6 mice (8–10 weeks old, 20–22 g) were obtained from the Animal Experiment Center of Hunan University of Medicine and housed under specific pathogen-free conditions. After one week of acclimatization, the mice were randomly assigned to seven groups (*n* = 10 per group): control group (control, received an equal volume of distilled water); high-dose apigenin group (Api-H, 50 mg kg^−1^); LPS model group (LPS, intratracheal instillation of 10 mg kg^−1^ LPS); LPS + low-dose apigenin group (LPS + API-L, 10 mg kg^−1^ LPS + 25 mg kg^−1^ apigenin); LPS + high-dose apigenin group (LPS + API − H, 10 mg kg^−1^ LPS + 50 mg kg^−1^ apigenin); LPS + ferroptosis inhibitor group (LPS + Fer-1, 10 mg kg^−1^ LPS + 10 mg kg^−1^ Fer-1); and LPS + high-dose apigenin + ferroptosis inducer group (LPS + API-H + Erastin, 10 mg kg^−1^ LPS + 50 mg kg^−1^ apigenin + 15 mg kg^−1^ Erastin). All treatments were administered *via* intraperitoneal injection 1 h prior to LPS challenge and continued once daily for three consecutive days. Samples were collected 24 h after the final LPS administration for subsequent analyses. The apigenin doses were selected based on preliminary dose–response experiments.

### 2.3. Cell culture and treatment

The BEAS-2B human bronchial epithelial cell line used in this study was obtained from the Cell Bank of the Chinese Academy of Sciences (Shanghai, China). Cells were cultured in RPMI-1640 medium containing 10% fetal bovine serum and 1% penicillin-streptomycin, under standard conditions of 37 °C with 5% CO_2_ in a humidified incubator. Prior to experimentation, cells in the logarithmic growth phase were plated and allowed to attach for 24 h. For intervention studies, the experimental setup comprised five groups: untreated controls; LPS-challenged cells (10 µg mL^−1^ LPS); LPS plus the ferroptosis inhibitor Fer-1 (10 µg mL^−1^ LPS + 5 µM Fer-1); LPS plus apigenin (10 µg mL^−1^ LPS + 40 µM apigenin); and LPS plus apigenin combined with the ferroptosis inducer Erastin (10 µg mL^−1^ LPS + 40 µM apigenin + 10 µM Erastin). Each treatment condition was performed in triplicate wells, and all interventions were maintained for 24 h. Following this period, both cell pellets and culture supernatants were harvested for subsequent analyses. Apigenin and Erastin concentrations were chosen according to preliminary cytotoxicity and efficacy tests.

### 2.4. Cell viability assay

To evaluate the potential cytotoxicity of apigenin toward BEAS-2B cells, the CCK-8 assay was employed. Cells at logarithmic growth stage were seeded into 96-well plates and incubated for 24 h to allow attachment. Subsequently, the culture medium was replaced with fresh medium containing apigenin at graded concentrations (2.5, 5, 10, 20, and 40 µM), whereas control wells received an equivalent volume of medium without the compound. After a 24 h exposure period, 10 µL of CCK-8 solution was added to each well, followed by a further 2 h incubation in the dark. Absorbance was read at 450 nm using a microplate reader, and cell viability percentages were calculated relative to the control group.

### 2.5. Histological staining

Tissues (lung, heart, liver, spleen, and kidney) collected from mice were fixed in 4% paraformaldehyde for 24 h, then dehydrated through a graded ethanol series, cleared with xylene, and embedded in paraffin blocks. Sections were cut at 4 µm thickness and subsequently stained with hematoxylin and eosin (H&E). The staining procedure consisted of deparaffinization, rehydration, hematoxylin staining for 5 min, differentiation in 1% hydrochloric acid in ethanol, bluing in ammonia water, eosin staining for 2 min, dehydration, clearing, and final mounting with neutral balsam. Histopathological changes were examined under a light microscope. Lung injury was semi-quantitatively assessed using a scoring system based on inflammatory cell infiltration, alveolar wall thickening, and structural disruption.

### 2.6. Biochemical assays

Lung tissue homogenates and BEAS-2B cell lysates were collected for the measurement of oxidative stress and ferroptosis-related parameters. SOD activity, GSH content, MDA levels, MPO activity, and Fe^2+^ concentrations were determined using commercial kits according to the manufacturers' protocols, employing xanthine oxidase, dithiodinitrobenzoic acid, thiobarbituric acid, colorimetric, and spectrophotometric methods, respectively. For inflammatory cytokine analysis, BALF, plasma, and cell culture supernatants were collected. Levels of IL-6, IL-1β, TNF-α, KL-6, and C-reactive protein (CRP) were quantified using corresponding ELISA kits. Absorbance was measured at the appropriate wavelengths using a microplate reader, and concentrations were calculated from standard curves.

### 2.7. Immunohistochemistry

For immunohistochemical analysis, paraffin-embedded lung tissue sections were subjected to deparaffinization and rehydration. Endogenous peroxidase activity was quenched by treatment with 3% H_2_O_2_. Antigen retrieval was performed using citrate buffer, followed by blocking with 5% bovine serum albumin (BSA) for 1 h at ambient temperature. Sections were then incubated overnight at 4 °C with primary antibodies targeting 4-HNE, GPX4, and SLC7A11, each diluted 1 : 200. After rewarming, the sections were incubated with HRP-conjugated secondary antibodies (1 : 500 dilution) for 1 h at room temperature. Immunoreactivity was developed using diaminobenzidine (DAB) as the chromogen, and nuclei were counterstained with hematoxylin. Finally, sections were dehydrated, cleared, and mounted for microscopic evaluation and image acquisition.

### 2.8. Fluorescence staining

To evaluate intracellular Fe^2+^ levels, mitochondrial reactive oxygen species (mtROS), and lipid peroxidation, BEAS-2B cells were stained with FerroOrange, MitoSOX Red, or C11-BODIPY, respectively, according to the manufacturers' instructions. After treatment, cells were washed with PBS, and staining was conducted for 30 min at 37 °C in the dark. Following removal of excess dye, fluorescence images were acquired using a laser scanning confocal microscope.

### 2.9. Western blot analysis

Total proteins were extracted from lung tissues and BEAS-2B cells using RIPA lysis buffer. Protein concentrations were determined using the BCA assay and normalized. Protein samples were separated by SDS-PAGE and transferred onto PVDF membranes. Membranes were blocked with 5% non-fat milk for 1 h and incubated overnight at 4 °C with primary antibodies against p65, p-p65, IκBα, p-IκBα, JAK2, p-JAK2, STAT3, GPX4, SLC7A11, IL-6, IL-1β, TNF-α, and β-actin. Following washing with TBST, the membranes were incubated with HRP-conjugated secondary antibodies for 1 h at room temperature. Protein bands were detected using an enhanced chemiluminescence (ECL) reagent and captured with a gel documentation system. Band intensities were analyzed using ImageJ software, and relative protein expression levels were normalized to β-actin.

### 2.10. RT-qPCR

Total RNA was extracted from lung tissues and BEAS-2B cells using TRIzol reagent. RNA concentration and purity were assessed spectrophotometrically, and complementary DNA (cDNA) was synthesized by reverse transcription according to the manufacturer's instructions. Real-time quantitative PCR (RT-qPCR) was performed using cDNA as a template with gene-specific primers (primer sequences are listed in Table S1). β-Actin served as the internal reference gene. The thermal cycling protocol consisted of an initial denaturation at 95 °C for 3 min, followed by 40 cycles of denaturation at 95 °C for 10 s and annealing/extension at 60 °C for 30 s. Relative mRNA expression levels of IL-6, IL-1β, TNF-α, GPX4, and SLC7A11 were quantified using the 2^−ΔΔCt^ method, with β-actin serving as the internal reference gene.

### 2.11. Network pharmacology analysis

Potential targets of apigenin were retrieved from PharmMapper, SwissTargetPrediction, SEA, and ChEMBL databases. After removing duplicates, the apigenin target set was obtained. Concurrently, disease targets associated with lung injury were retrieved from OMIM, GeneCards, and TTD databases. Duplicate entries were removed to generate the lung injury-related disease target set. The intersection of apigenin targets and lung injury targets was obtained using Venny 2.1.0, yielding 194 common targets.

The 194 common targets were submitted to the STRING database to construct a protein–protein interaction (PPI) network, with the species limited to *Homo sapiens* and a confidence score threshold of ≥0.4. The resulting network data were then imported into Cytoscape (version 3.10.1) for visualization, and core targets were identified based on their degree values. To further explore the biological functions of these common targets, Gene Ontology (GO) enrichment analysis and Kyoto Encyclopedia of Genes and Genomes (KEGG) pathway enrichment analysis were performed using the DAVID database. Detailed website information for all databases and tools used in this section is provided in Table S2.

### 2.12. Molecular docking

Crystal structures of ten core target proteins were downloaded from the Protein Data Bank (PDB). Water molecules and original ligands were removed, and structures were optimized using PyMOL software. The two-dimensional structure of apigenin was drawn using ChemDraw and converted into a three-dimensional conformation. Molecular docking was performed using AutoDock Vina software to evaluate the binding affinity between apigenin and the target proteins, with lower binding energy indicating more stable binding. The top three target proteins with the lowest binding energies (MMP9, EGFR, and ESR1) were selected for detailed interaction analysis. PyMOL and LigPlot + software were used to visualize and analyze the interaction patterns, including hydrogen bonds and hydrophobic interactions, between apigenin and the active sites of these proteins. See Table S2 for the corresponding website information.

### 2.13. Molecular dynamics simulation

The complexes formed between apigenin and the three core target proteins (MMP9, EGFR, and ESR1) were subjected to 100 ns molecular dynamics simulations using GROMACS software. The GROMOS 54a7 force field was employed, and the system was solvated with TIP3P water molecules. Na^+^ and Cl^−^ ions were added to neutralize the system. Energy minimization was performed to eliminate unrealistic atomic contacts, followed by equilibration under NVT and NPT ensembles to maintain a constant temperature of 300 K and pressure of 1 bar. Production simulations were run for 100 ns, and trajectory files were extracted for subsequent analysis. Various parameters, including root mean square deviation (RMSD), radius of gyration (*R*_g_), number of hydrogen bonds, solvent accessible surface area (SASA), and root mean square fluctuation (RMSF), were calculated using GROMACS built-in tools. In addition, free energy landscapes (FEL) were constructed with the gmx sham tool to evaluate conformational stability and assess the reliability of the binding modes of the apigenin-target complexes at the molecular level. Software details are summarized in Table S2.

### 2.14. Statistical analysis

GraphPad Prism 10.1.2 was used for all statistical analyses. Results are reported as mean ± standard deviation (SD). Intergroup differences were assessed using one-way analysis of variance (ANOVA) followed by the least significant difference (LSD) test for pairwise comparisons. A threshold of *P* < 0.05 was adopted to indicate statistical significance, while *P* < 0.01 and *P* < 0.001 were considered indicative of stronger statistical evidence. “ns” Denotes non-significant differences. A total of 10 mice were assigned to each group in the animal experiments. For cell-based assays, each treatment condition was performed in triplicate, and the experiments were independently repeated three times to confirm consistency.

## 3. Results

### 3.1. Network pharmacology analysis

**Fig. 1:**
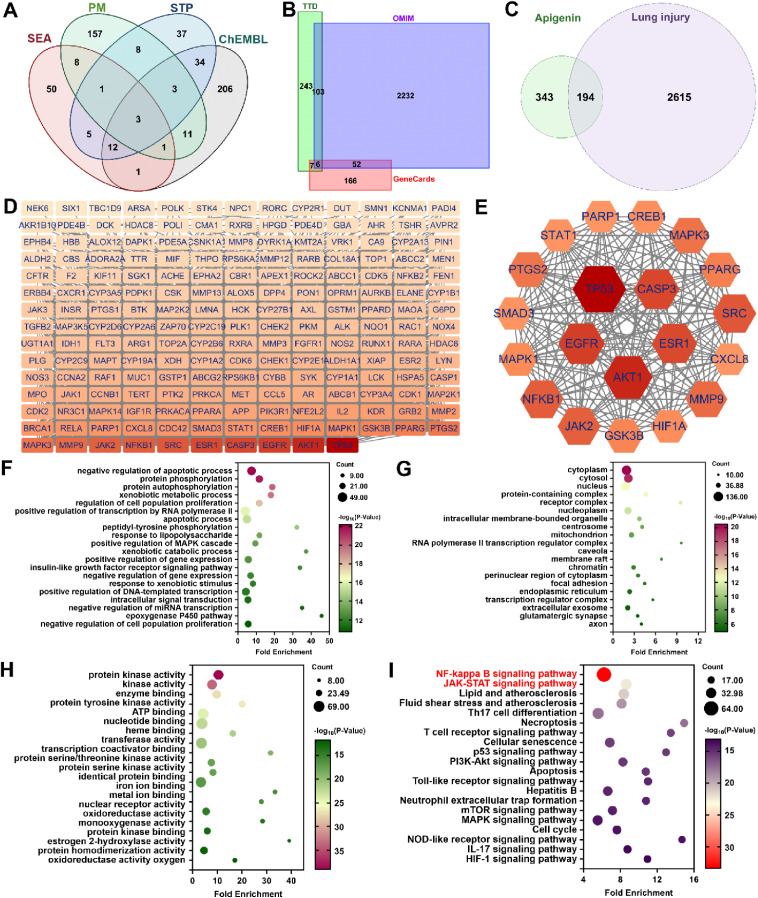
Network pharmacology analysis of potential targets of apigenin in the treatment of acute lung injury. (A) Collection of potential apigenin targets based on SEA, PharmMapper (PM), SwissTargetPrediction (STP), and ChEMBL databases. (B) Collection of lung injury-related targets based on TTD, OMIM, and GeneCards databases. (C) Venn diagram showing the intersection of apigenin targets and lung injury targets. (D) Visualization of the protein–protein interaction (PPI) network of apigenin-lung injury common targets. (E) Identification of core targets based on degree values. (F–H) Top 20 terms of GO enrichment analysis for common targets: (F) biological processes (BP); (G) cellular components (CC); (H) molecular functions (MF). (I) Top 20 pathways of KEGG pathway enrichment analysis for common targets.

To investigate the potential mechanisms underlying the therapeutic effects of apigenin in ALI, a network pharmacology approach was adopted to predict its relevant targets and associated signaling pathways. Target prediction using the PM, STP, SEA, and ChEMBL databases yielded 192, 103, 81, and 271 candidate targets, respectively, which after deduplication resulted in a total of 540 unique apigenin-associated targets. According to STP-based target distribution analysis, these targets were predominantly associated with proteases, kinases, and cytochrome P450 enzymes (Fig. S1). Meanwhile, lung injury-related targets were retrieved from the TTD, OMIM, and GeneCards databases, generating 359, 231, and 2393 entries, respectively. After removal of duplicates, 2818 unique disease-related targets were identified (Fig. 1A and B). Overlap analysis between the apigenin target set and the disease target set revealed 194 common targets, which likely represent key mediators of apigenin's protective action against ALI (Fig. 1C).

The 194 common targets were submitted to the STRING database for PPI network construction using a confidence score threshold of ≥0.4. The resulting network consisted of 193 nodes and 3131 edges, with an average node degree of 32.4 and an average local clustering coefficient of 0.556. A significant PPI enrichment (*p* < 0.05) indicated that these proteins interact more frequently than would be expected by chance, suggesting their functional interconnectedness (Fig. S2). The PPI data were subsequently imported into Cytoscape (version 3.10.1) for visualization, and core targets were selected based on degree centrality (Fig. 1D and E). The top ten targets by degree value were TP53, AKT1, EGFR, CASP3, ESR1, SRC, NFKB1, JAK2, MMP9, and MAPK3, pointing to their potential importance in apigenin's mechanism of action.

To further characterize the biological functions of these 194 shared targets, GO and KEGG enrichment analyses were performed using the DAVID database. As summarized in Fig. 1F–H, GO analysis showed that the targets were predominantly enriched in biological processes related to external stimuli responses, inflammatory responses, and redox regulation; cellular components such as the extracellular space, plasma membrane, and cytoplasm; and molecular functions including protein binding, ATP binding, and identical protein binding. KEGG pathway analysis indicated significant enrichment in several inflammation- and oxidative stress-related pathways, including NF-κB, JAK-STAT, PI3K-Akt, TNF, and HIF-1 signaling (Fig. 1I). Notably, the NF-κB and JAK-STAT pathways are closely associated with inflammatory regulation, supporting the hypothesis that apigenin may exert its anti-inflammatory effects through modulation of these cascades. Although other pathways were also enriched, subsequent experimental validation focused on the NF-κB and JAK-STAT pathways because of their central roles in inflammation and oxidative stress.

### 3.2. Molecular docking validation

**Fig. 2:**
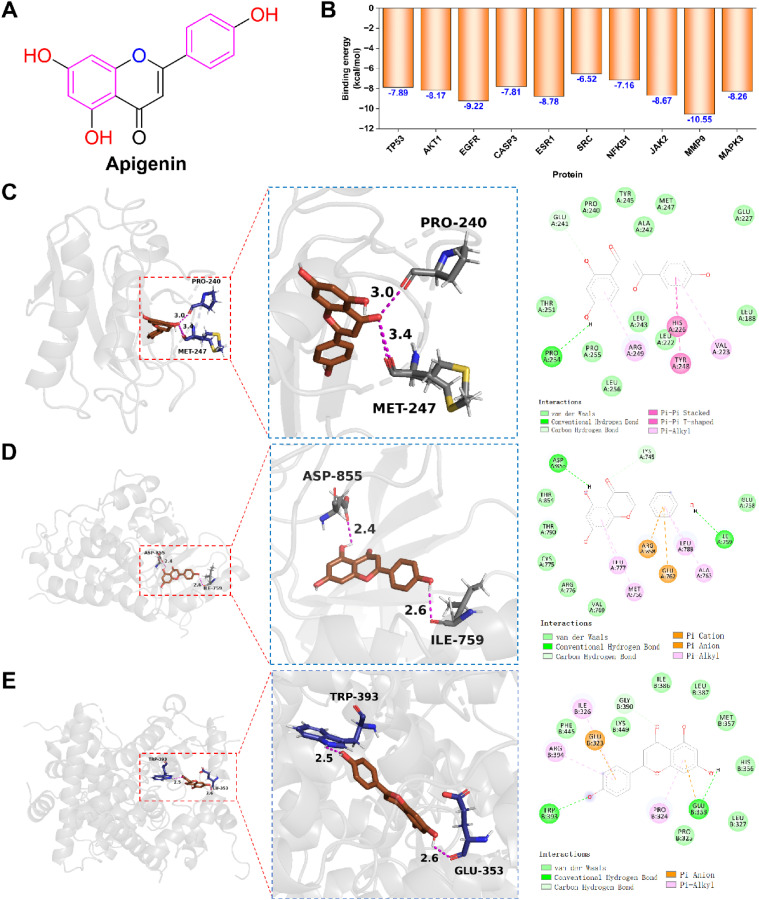
Molecular docking interaction analysis of apigenin with core target proteins. (A) Chemical structure of apigenin. (B) Binding energy histogram of molecular docking between apigenin and ten core target proteins. (C–E) Two-dimensional and three-dimensional visualization of molecular docking interactions between apigenin and (C) MMP9 (PDB: 4XCT), (D) EGFR (PDB: 8A2D), and (E) ESR1 (PDB: 8DU8).

To further validate the core targets predicted by network pharmacology, molecular docking technology was employed to evaluate the binding affinity between apigenin and the ten core target proteins. Molecular docking of apigenin (Fig. 2A) with the crystal structures of the ten core target proteins was performed using AutoDock Vina software, with lower binding energy indicating more stable ligand-receptor binding. As shown in Fig. 2B, the binding energies between apigenin and all ten core targets were less than −6.0 kcal mol^−1^, with the lowest binding energies observed for MMP9, EGFR, and ESR1 at −10.55 kcal mol^−1^, −9.22 kcal mol^−1^, and −8.78 kcal mol^−1^, respectively, indicating strong binding affinities between apigenin and these targets.

The top three target proteins with the lowest binding energies (MMP9, EGFR, and ESR1) were selected for detailed interaction mode analysis. Fig. 2C–E present the two-dimensional and three-dimensional molecular docking interaction diagrams of apigenin with MMP9, EGFR, and ESR1, respectively. The results demonstrated that apigenin primarily binds to the active sites of target proteins through hydrogen bonds and hydrophobic interactions. Specifically, apigenin formed hydrogen bonds with amino acid residues PRO-240 and MET-247 in the active site of MMP9; with ASP-855 and ILE-759 residues in EGFR; and with TRP-393 and GLU-353 residues in ESR1. These interaction patterns provide a structural basis for the stable binding of apigenin to these target proteins. Furthermore, Fig. S3 displays the two-dimensional molecular docking interaction diagrams of apigenin with the remaining seven core target proteins, while Fig. S4 shows the two-dimensional interaction diagrams of apigenin with all ten core target proteins. These results further confirm that apigenin can stably bind to core target proteins through multiple non-covalent interactions.

### 3.3. Molecular dynamics simulation validation

**Fig. 3:**
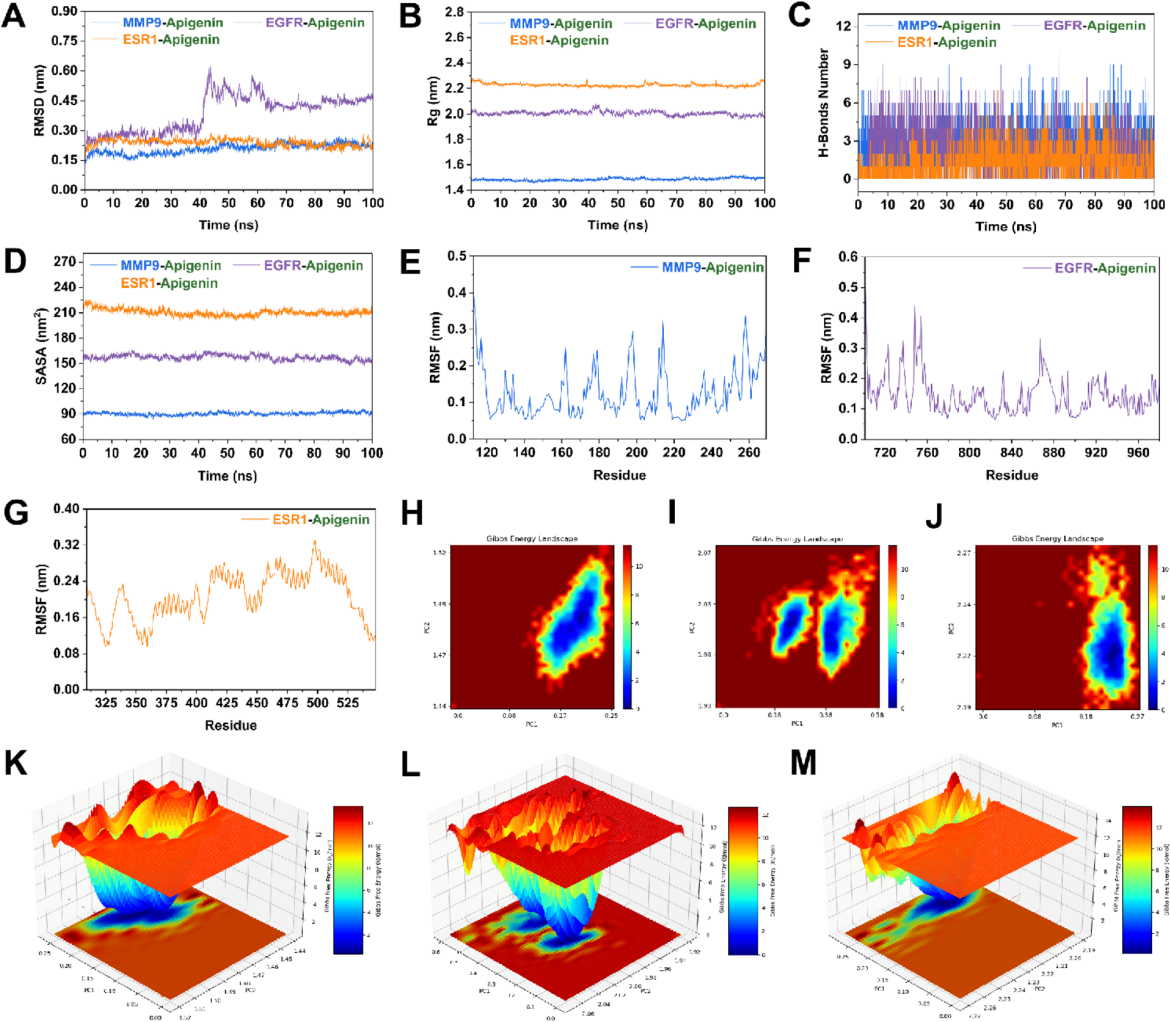
Molecular dynamics simulation analysis of apigenin complexes with core target proteins. (A–D) Time-dependent changes in RMSD, radius of gyration (*R*_g_), number of hydrogen bonds, and solvent accessible surface area (SASA) for apigenin complexes with MMP9, EGFR, and ESR1. (E–G) RMSF plots of the three complexes. (H–J) Two-dimensional free energy landscapes of the three complexes. (K–M) Three-dimensional free energy landscapes of the three complexes.

To comprehensively evaluate the stability of the binding modes between apigenin and the core target proteins MMP9, EGFR, and ESR1, 100 ns molecular dynamics simulations were performed on the three respective complexes to investigate their conformational changes and binding stability during dynamic processes. As shown in Fig. 3A, the RMSD values of the apigenin-MMP9, EGFR, and ESR1 complexes gradually stabilized during the simulation, fluctuating around 0.15 nm, 0.3 nm, and 0.45 nm, respectively, with relatively small amplitudes, indicating that all three complexes reached equilibrium states with stable conformations. The *R*_g_, which reflects protein structural compactness, remained relatively constant throughout the simulation for all three complexes, suggesting that apigenin binding did not induce overall structural loosening or excessive folding of the target proteins (Fig. 3B). Hydrogen bond analysis revealed that apigenin formed an average of three hydrogen bonds with MMP9, EGFR, and ESR1, and the number of hydrogen bonds remained stable throughout the simulation, indicating that hydrogen bonding interactions are important factors in maintaining complex stability (Fig. 3C). SASA analysis showed that the SASA values of the three complexes stabilized in the later stages of simulation without significant fluctuations, further confirming the conformational stability of the complexes (Fig. 3D).

Residue flexibility was assessed through RMSF analysis. The results demonstrated that after apigenin binding, the RMSF values of residues in the active site regions of MMP9, EGFR, and ESR1 were relatively low, indicating enhanced rigidity in these regions that facilitates stable binding, while loop regions distant from the binding sites exhibited higher flexibility, consistent with protein dynamics characteristics (Fig. 3E–G). FEL analysis was employed to examine the distribution of low-energy conformations of the complexes. As shown in the two-dimensional and three-dimensional FEL plots, all three complexes exhibited single, concentrated energy valleys, indicating that the complexes primarily remained in low-energy conformational states during the simulation with concentrated conformational spaces, further confirming the high stability of the apigenin-MMP9, EGFR, and ESR1 complexes (Fig. 3H–M).

### 3.4. Apigenin attenuates LPS-induced acute lung injury in mice

**Fig. 4:**
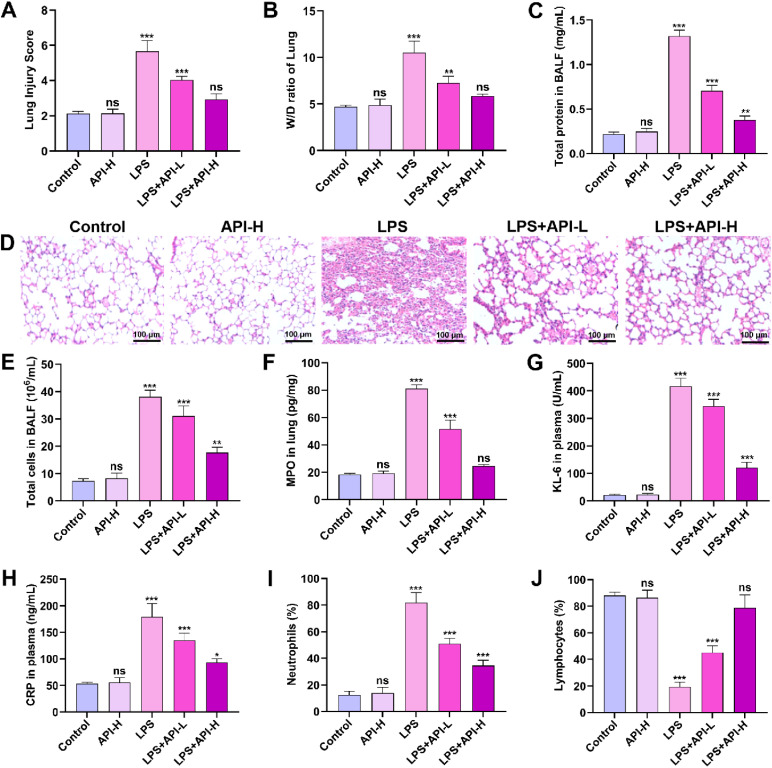
Effects of apigenin on pulmonary histopathology and related parameters in mice with acute lung injury. (A) Lung injury scores. (B) Lung wet-to-dry weight (W/D) ratio. (C) Total protein concentration in bronchoalveolar lavage fluid (BALF). (D) Representative images of H&E-stained lung tissue sections (scale bar = 100 µm). (E) Total cell count in BALF. (F) Myeloperoxidase (MPO) activity in lung tissue. (G) Plasma KL-6 levels. (H) Plasma C-reactive protein (CRP) levels. (I) Percentage of neutrophils in peripheral blood. (J) Percentage of lymphocytes in peripheral blood. *P < 0.05, **P < 0.01, ***P < 0.001 *vs.* control group; ns indicates no significant difference.

To assess whether apigenin protects against acute lung injury, a mouse model was generated *via* intratracheal LPS administration, followed by treatment with apigenin at graded doses. As illustrated in Fig. 4, LPS-challenged mice showed pronounced increases in lung injury scores, lung W/D ratio, and total protein levels in BALF relative to controls, indicative of pulmonary edema and alveolar-capillary barrier disruption. Apigenin treatment dose-dependently reversed these abnormalities (Fig. 4A–C). H&E staining revealed that LPS exposure led to substantial inflammatory cell infiltration, alveolar wall thickening, and distorted alveolar architecture, all of which were markedly alleviated by apigenin, especially at the higher dose (Fig. 4D). In addition, apigenin administration significantly reduced total BALF cell counts and decreased lung MPO activity, suggesting attenuation of LPS-induced pulmonary inflammatory infiltration and neutrophil activation (Fig. 4E and F). Consistent with these observations, plasma levels of KL-6 and CRP were significantly lower in apigenin-treated mice than in those receiving LPS alone (Fig. 4G and H). Peripheral blood analysis further indicated that apigenin counteracted the LPS-induced increase in neutrophil percentage and decrease in lymphocyte percentage (Fig. 4I and J). Taken together, these findings demonstrate that apigenin exerts dose-dependent protective effects against LPS-induced ALI, as evidenced by improved pulmonary histopathology and reduced inflammatory cell infiltration.

### 3.5. Apigenin suppresses LPS-induced pulmonary inflammatory responses

**Fig. 5:**
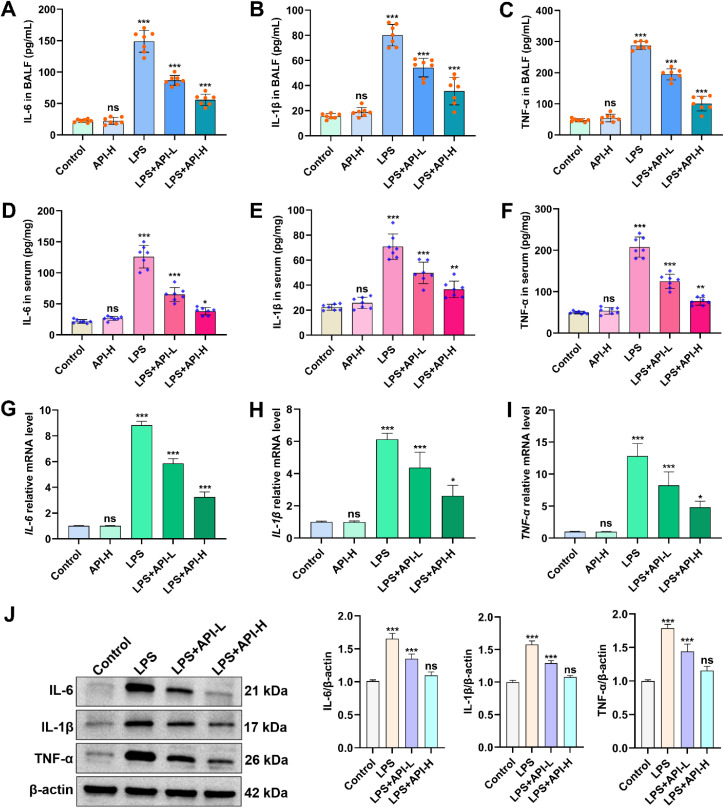
Effects of apigenin on inflammatory cytokine expression in mice with acute lung injury. (A–C) Concentrations of IL-6, IL-1β, and TNF-α in bronchoalveolar lavage fluid (BALF) (*n* = 7). (D–F) Concentrations of IL-6, IL-1β, and TNF-α in plasma (*n* = 7). (G–I) Relative mRNA expression levels of IL-6, IL-1β, and TNF-α in lung tissue (*n* = 7). (J) Protein expression levels of IL-6, IL-1β, and TNF-α in lung tissue (*n* = 3). *P < 0.05, **P < 0.01, ***P < 0.001 *vs.* control group; ns indicates no significant difference.

To evaluate the effect of apigenin on LPS-induced pulmonary inflammation, levels of inflammatory cytokines in BALF and plasma, as well as their mRNA and protein expression in lung tissue, were examined. As shown in Fig. 5, compared with the control group, administration of high-dose apigenin alone did not significantly alter any of the inflammatory parameters, indicating that apigenin itself does not provoke inflammatory responses. In the LPS model group, the concentrations of IL-6, IL-1β, and TNF-α in BALF were markedly elevated, and similar increases were observed in plasma (Fig. 5A–F). Furthermore, LPS stimulation significantly upregulated the mRNA and protein expression levels of these inflammatory cytokines in lung tissue, confirming the successful establishment of pulmonary and systemic inflammation (Fig. 5G–J). Compared with the LPS model group, apigenin pretreatment, particularly at the high dose, significantly reduced the levels of IL-6, IL-1β, and TNF-α in both BALF and plasma and downregulated their corresponding mRNA and protein expression in lung tissue (Fig. 5). Although the low-dose apigenin group exhibited a trend toward inhibition, some parameters did not reach statistical significance. These findings demonstrate that apigenin effectively suppresses LPS-induced pulmonary and systemic inflammatory responses in a dose-dependent manner.

### 3.6. Apigenin modulates the NF-κB/JAK2-STAT3 signaling pathway

**Fig. 6:**
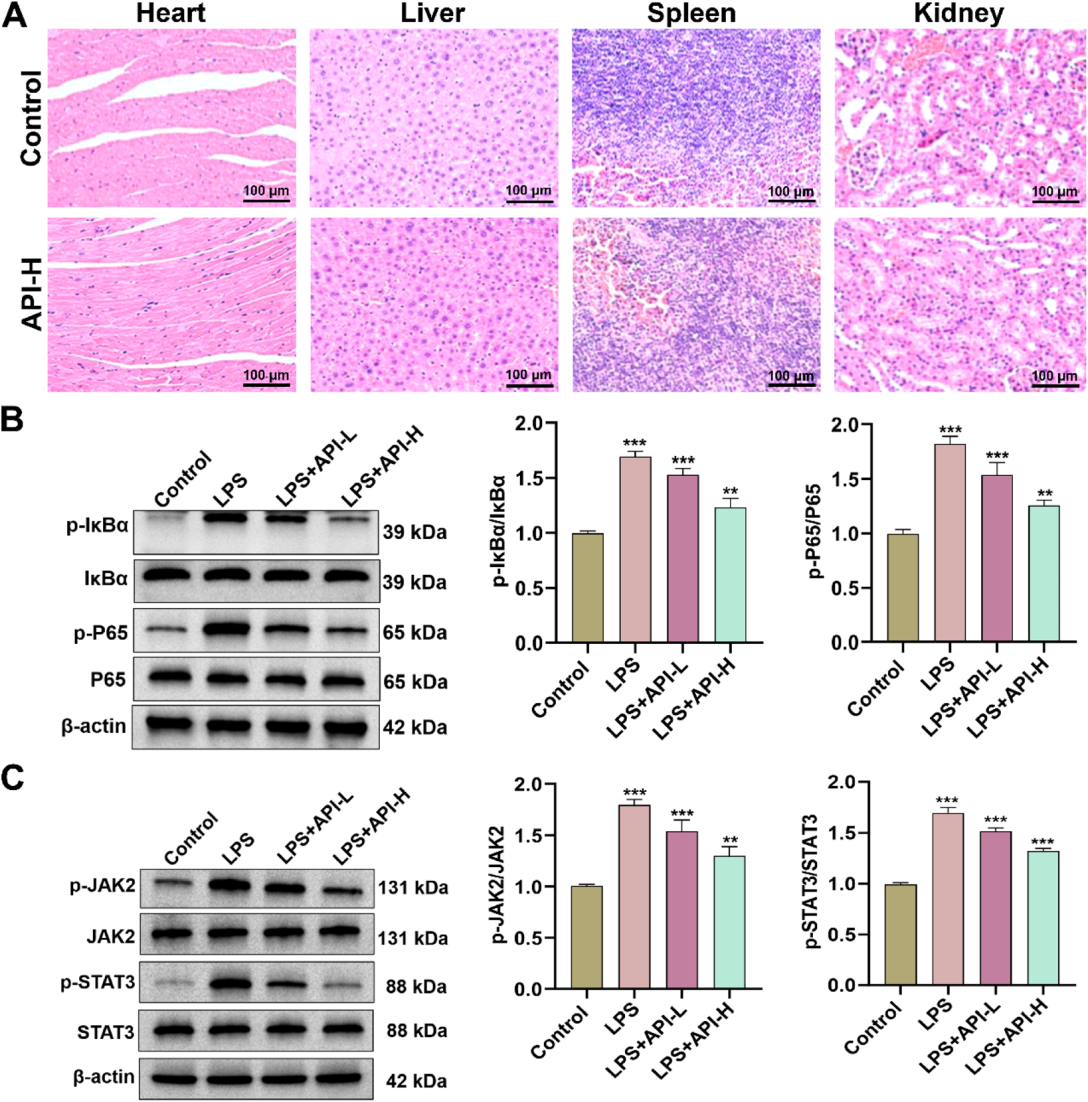
Effects of apigenin on *in vivo* safety and NF κB/JAK2 STAT3 pathway protein expression in lung tissue of mice with acute lung injury. (A) H&E staining of heart, liver, spleen, and kidney sections for *in vivo* safety assessment of apigenin (scale bar = 100 µm). (B) Expression levels of NF κB signaling pathway-related proteins (p65, p p65, IκBα, p IκBα). (C) Expression levels of JAK2 STAT3 signaling pathway-related proteins (JAK2, p JAK2, STAT3, p STAT3). *P < 0.05, **P < 0.01, ***P < 0.001 *vs.* control group; ns indicates no significant difference.

To verify the pathway predictions derived from network pharmacology, we initially evaluated the *in vivo* safety profile of apigenin, followed by an assessment of its impact on the NF-κB and JAK2-STAT3 signaling cascades. H&E staining of heart, liver, spleen, and kidney sections from control mice and those receiving high-dose apigenin revealed no discernible morphological alterations: myocardial fibers displayed regular arrangement, hepatocytes maintained clear architecture, splenic corpuscles appeared unremarkable, and glomerular and tubular structures in the kidney remained intact (Fig. 6A). These observations support the favorable *in vivo* safety of apigenin at the administered high dose.

Western blot analysis was conducted to evaluate the expression of key proteins within the NF-κB and JAK2-STAT3 pathways in lung tissue. As depicted in Fig. 6B, LPS challenge significantly elevated the p-p65/p65 and p-IκBα/IκBα ratios relative to the control group, indicative of NF-κB pathway activation. Pretreatment with apigenin reduced these ratios in a dose-dependent fashion, with the most pronounced inhibition observed at the higher dose. Similarly, LPS exposure markedly increased the p-JAK2/JAK2 and p-STAT3/STAT3 ratios in lung tissue, reflecting activation of the JAK2-STAT3 pathway (Fig. 6C). Apigenin treatment dose-dependently suppressed these phosphorylation events, with the high-dose group again exhibiting the strongest inhibitory effect.

### 3.7. Apigenin inhibits LPS-induced oxidative stress and ferroptosis in lung tissue

**Fig. 7:**
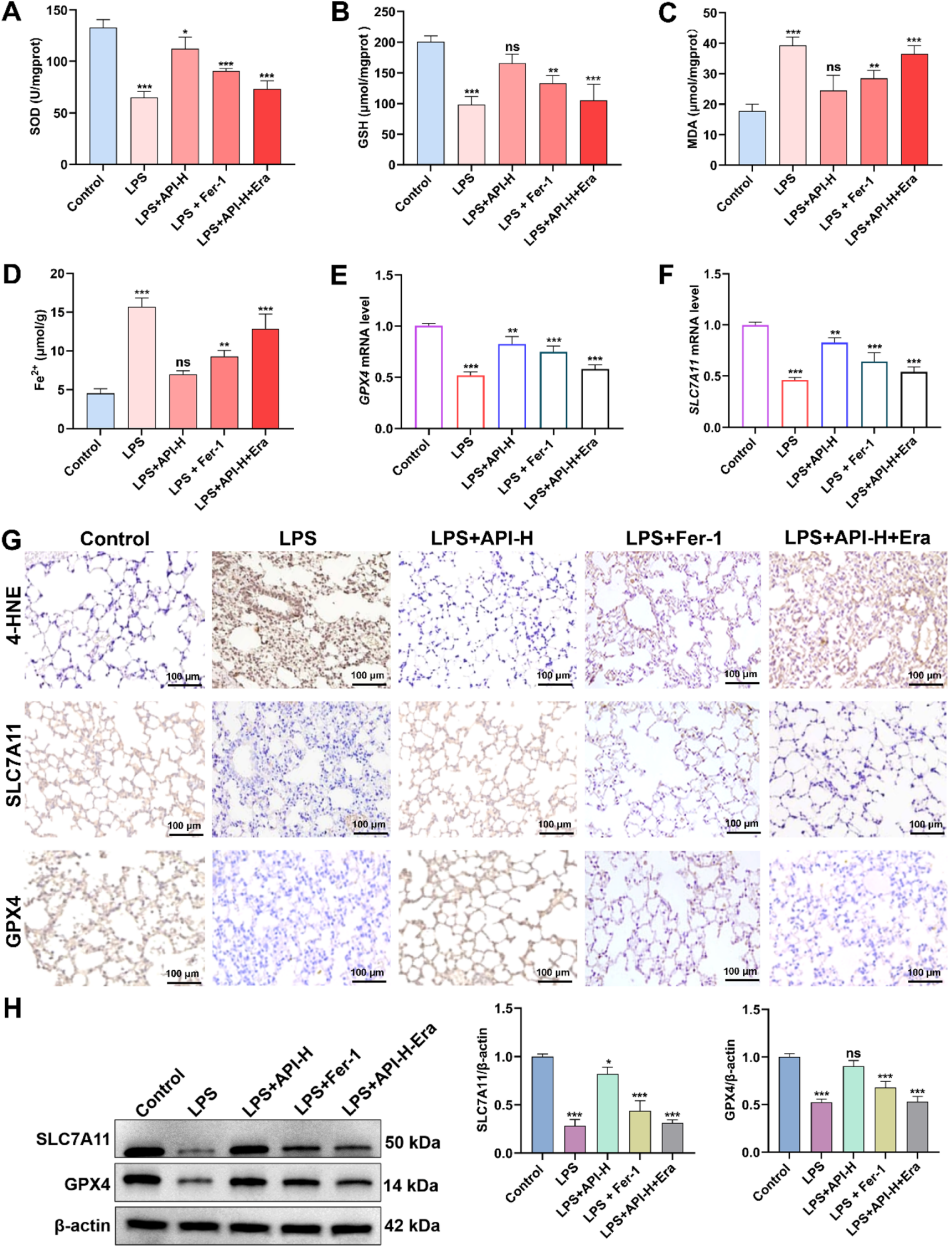
Effects of apigenin on oxidative stress and ferroptosis-related indicators in lung tissue of mice with acute lung injury. (A–D) Levels of SOD, GSH, MDA, and Fe^2+^ in lung tissue (*n* = 7). (E and F) mRNA expression levels of GPX4 and SLC7A11 in mouse lung tissue (*n* = 7). (G) Immunohistochemical staining of 4 HNE, GPX4, and SLC7A11 in lung tissue (*n* = 3, Scale bar = 100 µm). (H) Protein expression levels of GPX4 and SLC7A11 in mouse lung tissue (*n* = 3). *P < 0.05, **P < 0.01, ***P < 0.001 *vs.* control group; ns indicates no significant difference.

To examine the influence of apigenin on oxidative stress and ferroptosis in the context of LPS-induced ALI, we assessed oxidative stress parameters and key ferroptosis-related molecules in lung tissue, with validation performed using the ferroptosis inhibitor Fer-1 and inducer Erastin. As presented in Fig. 7A–C, the LPS model group showed marked reductions in SOD activity and GSH content, alongside a substantial increase in MDA levels relative to the control group, consistent with LPS-triggered oxidative injury. Administration of either the ferroptosis inhibitor Fer-1 or high-dose apigenin significantly enhanced SOD activity and GSH content while lowering MDA levels compared with the LPS group, with apigenin displaying a slightly more pronounced protective effect than Fer-1. Notably, co-treatment with the ferroptosis inducer Erastin partially reversed these protective effects, as evidenced by decreased SOD activity and GSH content and increased MDA levels.

Further analysis of ferroptosis-related indicators revealed that Fe^2+^ levels were significantly elevated in lung tissue from the LPS model group; this accumulation was markedly attenuated by Fer-1 or apigenin intervention, whereas the combination of Erastin with apigenin blunted the suppressive effect of apigenin on Fe^2+^ (Fig. 7D). As illustrated in Fig. 7E and F, LPS stimulation led to pronounced downregulation of GPX4 and SLC7A11 mRNA expression, an effect that was significantly reversed by Fer-1 or apigenin treatment, while the addition of Erastin counteracted this upregulation. Immunohistochemical staining results demonstrated that the LPS group exhibited markedly enhanced 4-HNE positive staining and reduced GPX4 and SLC7A11 positive staining in lung tissue; Fer-1 or apigenin intervention attenuated 4-HNE staining and enhanced GPX4 and SLC7A11 staining; however, Erastin combined with apigenin partially counteracted these effects of apigenin (Fig. 7G). Western blot results further confirmed that LPS downregulated the protein expression of GPX4 and SLC7A11, which was restored by Fer-1 or apigenin treatment, while Erastin combined with apigenin again decreased the expression of both proteins (Fig. 7H).

### 3.8. Apigenin attenuates LPS-induced cell injury and inflammatory response in BEAS-2B cells

**Fig. 8:**
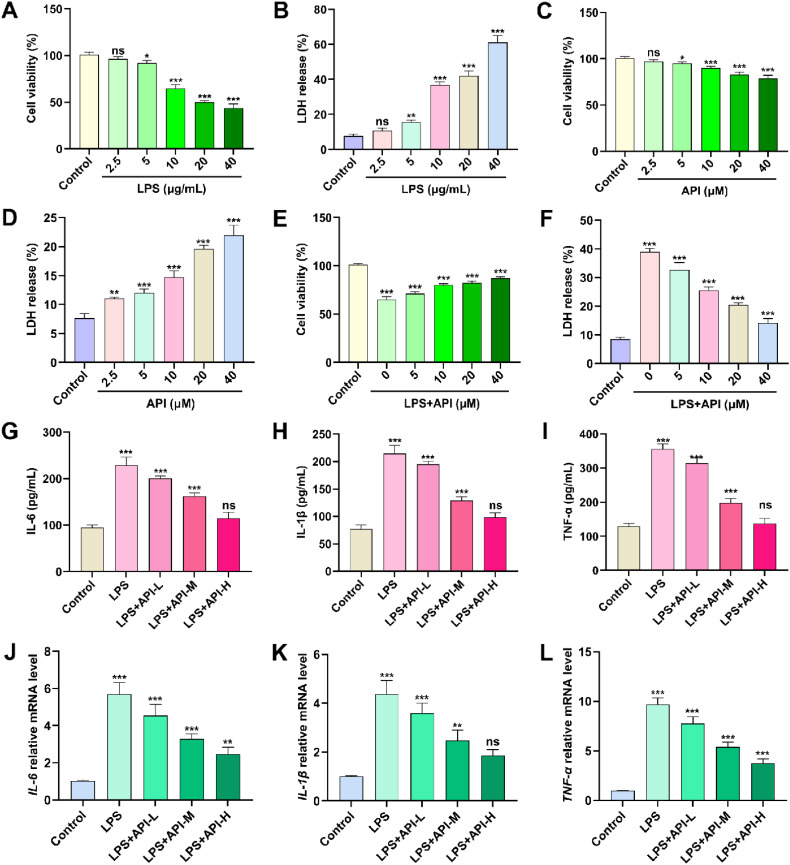
Effects of apigenin on cell viability, cytotoxicity, and inflammatory cytokine expression in LPS stimulated BEAS 2B cells. (A) Effect of different concentrations of LPS on BEAS 2B cell viability. (B) Effect of different concentrations of LPS on LDH release from BEAS 2B cells. (C) Effect of different concentrations of apigenin on BEAS 2B cell viability. (D) Effect of different concentrations of apigenin on LDH release from BEAS 2B cells. (E) Restoration effect of apigenin on LPS induced decrease in BEAS 2B cell viability. (F) Restoration effect of apigenin on LPS induced increase in LDH release from BEAS 2B cells. (G–I) Levels of IL 6, IL 1β, and TNF α in cell culture supernatants (L: 10 µM; M: 20 µM; H: 40 µM). (J–L) mRNA expression levels of IL 6, IL 1β, and TNF α in cells. *P < 0.05, **P < 0.01, ***P < 0.001 *vs.* control group; ns indicates no significant difference.

Based on the *in vivo* anti-inflammatory effects observed, we next examined whether apigenin exerts direct protective actions on bronchial epithelial cells using an *in vitro* model of LPS-induced injury in BEAS-2B human bronchial epithelial cells. Experimental conditions were first optimized. CCK-8 assays revealed that LPS reduced cell viability in a concentration-dependent manner; exposure to 10 µg mL^−1^ LPS for 24 h decreased viability to approximately 65% of that in control cells, and this concentration was therefore selected for subsequent experiments (Fig. 8A). LDH release measurements showed a trend consistent with the changes in cell viability (Fig. 8B). Cytotoxicity assessment indicated that apigenin alone, across concentrations of 2.5–40 µM, did not significantly affect BEAS-2B cell viability or LDH release, confirming its lack of cytotoxic effects within this range (Fig. 8C and D). Based on these findings, apigenin concentrations of 10, 20, and 40 µM were designated as low, medium, and high intervention doses, respectively, for subsequent studies. As illustrated in Fig. 8E and F, LPS challenge significantly decreased cell viability and increased LDH release, effects that were reversed by apigenin pretreatment in a concentration-dependent manner, with the most pronounced protection observed at 40 µM.

Further analysis of inflammatory cytokine expression revealed that LPS stimulation markedly elevated the protein levels of IL-6, IL-1β, and TNF-α in the culture supernatant, whereas apigenin pretreatment dose-dependently suppressed the release of these cytokines (Fig. 8G–I). Correspondingly, RT-qPCR analysis showed that LPS significantly upregulated the mRNA transcript levels of IL-6, IL-1β, and TNF-α, and apigenin intervention concentration-dependently reduced their expression, with the high-dose group exhibiting the most marked inhibitory effect (Fig. 8J–L). Collectively, these results indicate that apigenin acts directly on BEAS-2B cells to mitigate LPS-induced cytotoxicity and suppress the production of pro-inflammatory cytokines at both the transcriptional and translational levels.

### 3.9. Apigenin regulates cellular inflammation and the NF-κB/JAK2-STAT3 pathway in BEAS-2B cells

**Fig. 9:**
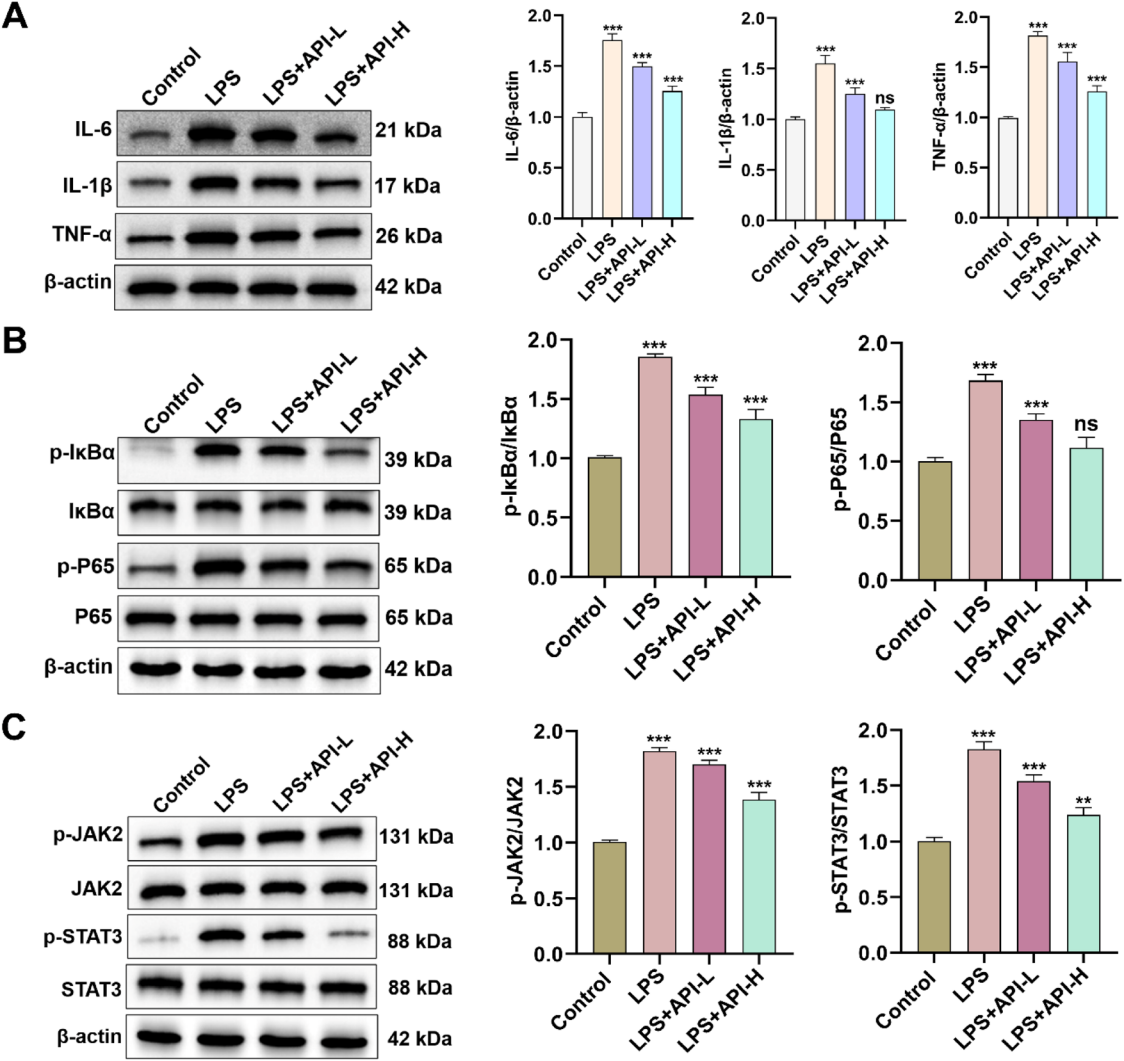
Effects of apigenin on inflammatory cytokine and signaling pathway protein expression in LPS induced BEAS 2B cells. (A) Protein expression levels of IL 6, IL 1β, and TNF α in cells. (B) Expression of NF κB signaling pathway-related proteins. (C) Expression of JAK2 STAT3 signaling pathway-related proteins. *P < 0.05, **P < 0.01, ***P < 0.001 *vs.* control group; ns indicates no significant difference.

To further explore the molecular mechanisms underlying the anti-inflammatory activity of apigenin at the cellular level, we assessed the phosphorylation status of key components of the NF-κB and JAK2-STAT3 pathways, alongside the expression of inflammatory cytokines, in BEAS-2B cells. As depicted in Fig. 9A, LPS stimulation led to a marked upregulation of IL-6, IL-1β, and TNF-α protein levels, consistent with the *in vivo* observations. Treatment with apigenin reversed these changes in a concentration-dependent manner, with the greatest inhibition occurring at 40 µM. Examination of the NF-κB pathway revealed that LPS treatment markedly increased the p-p65/p65 and p-IκBα/IκBα ratios, indicative of pathway activation. Apigenin intervention concentration-dependently suppressed the phosphorylation of both p65 and IκBα, reaching statistical significance at 40 µM (Fig. 9B). Similarly, analysis of the JAK2-STAT3 pathway showed that LPS challenge significantly elevated the p-JAK2/JAK2 and p-STAT3/STAT3 ratios, and apigenin treatment dose-dependently reduced these ratios, indicating effective inhibition of JAK2 and STAT3 phosphorylation (Fig. 9C).

### 3.10. Apigenin inhibits LPS-induced oxidative stress and ferroptosis in BEAS-2B cells

**Fig. 10:**
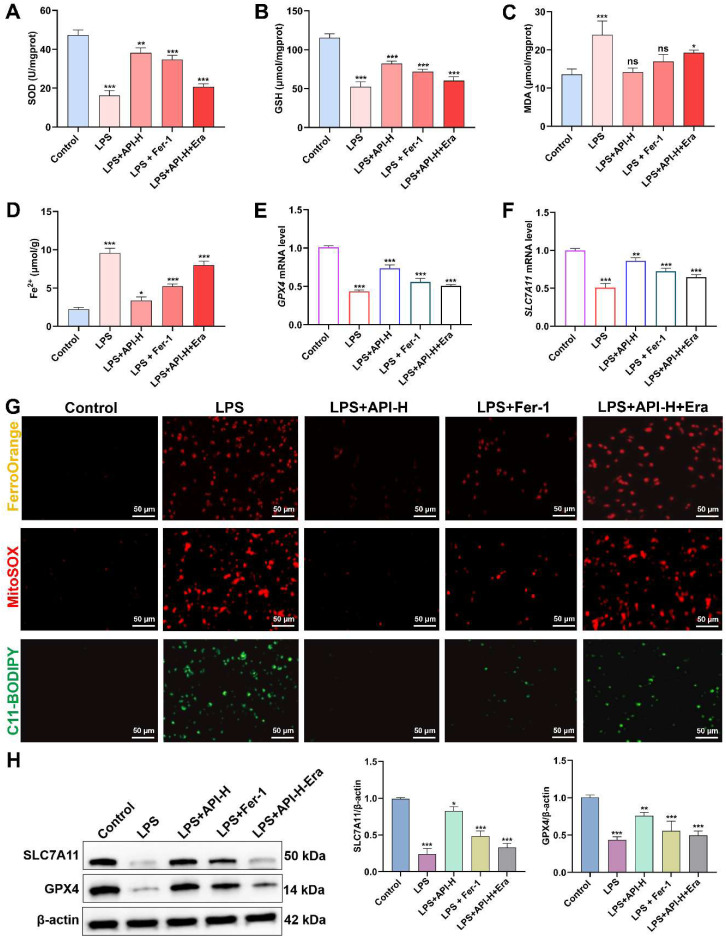
Effects of apigenin on oxidative stress and ferroptosis-related indicators in LPS-induced BEAS-2B cells. (A–D) Levels of SOD, GSH, MDA, and Fe^2+^ in cells (*n* = 7). (E and F) mRNA expression levels of GPX4 and SLC7A11 in cells (*n* = 7). (G) Fluorescence staining with FerroOrange, MitoSOX, and C11-BODIPY for detection of intracellular Fe^2+^ levels, mitochondrial reactive oxygen species (mtROS), and lipid peroxidation levels, respectively. (H) Protein expression levels of GPX4 and SLC7A11 in cells (*n* = 3). *P < 0.05, **P < 0.01, ***P < 0.001 *vs.* control group; ns indicates no significant difference.

To validate the anti-ferroptotic effects of apigenin observed *in vivo* and to investigate its direct actions at the cellular level, mechanistic experiments were conducted in BEAS-2B cells using the ferroptosis inhibitor Fer-1 and the inducer Erastin. As shown in Fig. 10A–C, LPS stimulation markedly reduced SOD activity and GSH content while increasing MDA levels, indicating the onset of oxidative stress injury. Treatment with either apigenin or Fer-1 significantly alleviated these oxidative stress parameters, with apigenin showing slightly greater protective effects than Fer-1. Notably, co-treatment with the ferroptosis inducer Erastin partially reversed the protective effects of apigenin, as reflected by decreased SOD and GSH levels and elevated MDA content.

Assessment of ferroptosis-related markers revealed that LPS significantly elevated intracellular Fe^2+^ levels, which were effectively reduced by apigenin or Fer-1 treatment; this effect was attenuated when Erastin was combined with apigenin (Fig. 10D). Similarly, LPS caused a marked downregulation of GPX4 and SLC7A11 mRNA expression, which was restored by apigenin or Fer-1, whereas the addition of Erastin counteracted this upregulation (Fig. 10E and F). Fluorescence staining further confirmed these findings (Fig. 10G). FerroOrange staining showed enhanced intracellular Fe^2+^ fluorescence in LPS-treated cells, which was diminished by apigenin or Fer-1. MitoSOX staining revealed increased mitochondrial superoxide levels in the LPS group, which were reduced following apigenin or Fer-1 intervention. C11-BODIPY staining indicated a higher ratio of oxidized to reduced fluorescence in LPS-challenged cells, indicative of lipid peroxidation, and this effect was reversed by apigenin or Fer-1. In all three assays, co-treatment with Erastin partially counteracted the protective effects of apigenin. Western blot analysis further confirmed that LPS downregulated GPX4 and SLC7A11 protein expression, which was restored by apigenin or Fer-1, while Erastin again reversed these effects (Fig. 10H).

## 4. Discussion

ALI and its more severe form, ARDS, continue to pose life-threatening challenges in clinical practice, with limited pharmacological interventions and persistently high mortality.^26^ Apigenin, a naturally occurring flavonoid found abundantly in fruits, vegetables, and various medicinal herbs, is well recognized for its diverse pharmacological properties, particularly its anti-inflammatory and antioxidant activities.^21,22^ In this work, we employed a combined approach integrating *in vivo* and *in vitro* experiments with network pharmacology, molecular docking, and molecular dynamics simulations to systematically explore the protective effects of apigenin against LPS-induced ALI. Our results provide the first evidence that apigenin confers pulmonary protection through coordinated regulation of the NF-κB/JAK2-STAT3 pathway alongside inhibition of ferroptosis and oxidative stress.

Mounting evidence points to dysregulated inflammation as a central driver in the pathogenesis of ALI.^27^ Upon LPS stimulation, multiple pro-inflammatory signaling cascades become activated, with the NF-κB and JAK2-STAT3 pathways considered particularly critical in this process.^28,29^ Activation of NF-κB promotes the transcriptional upregulation of pro-inflammatory cytokines such as IL-6, IL-1β, and TNF-α, whereas the JAK2-STAT3 axis further amplifies the inflammatory response.^30^ In line with these observations, our *in vivo* and *in vitro* experiments showed that apigenin pretreatment dose-dependently suppressed LPS-induced phosphorylation of p65 and IκBα, as well as that of JAK2 and STAT3, in both lung tissue and BEAS-2B cells. These findings indicate that apigenin exerts its anti-inflammatory effects by concurrently blocking the activation of the NF-κB and JAK2-STAT3 pathways, a dual-targeting strategy that aligns with the predictions from network pharmacology and suggests potential synergistic anti-inflammatory benefits.

Over the past several years, ferroptosis—a distinct mode of regulated cell death driven by iron-dependent lipid peroxidation—has gained increasing recognition as a critical pathological process in ALI.^31,32^ A key regulator of this process is GPX4, which safeguards membrane integrity by converting toxic lipid peroxides into non-toxic lipid alcohols.^33–35^ In our experiments, LPS challenge resulted in decreased GPX4 and SLC7A11 expression, elevated Fe^2+^ levels, and accumulation of lipid peroxidation markers (MDA and 4-HNE) in both lung tissue and BEAS-2B cells, confirming ferroptosis involvement in LPS-induced lung injury. Notably, apigenin treatment effectively restored GPX4 and SLC7A11 expression, reduced Fe^2+^ accumulation, and mitigated lipid peroxidation, with protective effects comparable to those of the ferroptosis inhibitor Fer-1. Furthermore, the ferroptosis inducer Erastin partially reversed these protective effects, reinforcing the conclusion that suppression of ferroptosis represents a key mechanism underlying the pulmonary protective activity of apigenin.

Accumulating evidence points to a bidirectional crosstalk between inflammation and ferroptosis. Activation of the NF-κB pathway promotes the release of inflammatory cytokines, which in turn can exacerbate oxidative stress and drive ferroptosis; conversely, lipid peroxides generated during ferroptosis may further amplify inflammatory signaling, thereby creating a self-reinforcing loop.^19,20,36,37^ By concurrently modulating the NF-κB/JAK2-STAT3 axis and inhibiting ferroptosis, apigenin may have the capacity to interrupt this detrimental cycle, leading to synergistic pulmonary protection. While our findings support this notion, additional mechanistic studies are needed to fully unravel the molecular interplay between these pathways. This work provides the first evidence that apigenin dually targets NF-κB/JAK2-STAT3 and GPX4/SLC7A11 to suppress inflammation and ferroptosis in ALI, as supported by Erastin rescue experiments, suggesting a novel mechanistic crosstalk.

The integration of network pharmacology with molecular docking and molecular dynamics simulations has become a widely adopted strategy for predicting and validating the mechanisms of action of natural products.^38,39^ Using this approach, we identified 194 potential targets of apigenin relevant to ALI, with KEGG enrichment analysis revealing significant enrichment of the NF-κB and JAK-STAT pathways. Molecular docking analyses demonstrated strong binding affinities between apigenin and core targets including MMP9, EGFR, and ESR1, and subsequent molecular dynamics simulations confirmed the stability of these interactions. Collectively, these computational findings not only corroborate the network pharmacology predictions but also provide valuable directions for future in-depth mechanistic investigations.

Several limitations should be acknowledged. First, although network pharmacology and molecular docking identified multiple potential targets of apigenin, direct binding interactions—such as between apigenin and MMP9 or EGFR—have not yet been experimentally validated using techniques such as cellular thermal shift assay (CETSA) or surface plasmon resonance (SPR). Second, while the involvement of ferroptosis was confirmed using Fer-1 and Erastin, the precise mechanisms linking the NF-κB/JAK2-STAT3 pathway to ferroptosis regulation remain incompletely understood. Future studies employing genetic knockdown or knockout of key molecules such as GPX4 or STAT3 in lung epithelial cells will be necessary to establish causal relationships.

## 5. Conclusion

In conclusion, this study integrated network pharmacology, molecular docking, and 100 ns molecular dynamics simulations to systematically predict the core targets and key signaling pathways involved in the protective effects of apigenin against ALI. Building on these computational predictions, subsequent *in vivo* and *in vitro* experiments validated that apigenin dose-dependently suppressed the phosphorylation of NF-κB and JAK2-STAT3 pathways, upregulated GPX4 and SLC7A11 expression, reduced Fe^2+^ accumulation and lipid peroxidation, thereby coordinately inhibiting inflammatory responses and ferroptosis. The mutual corroboration between computational predictions and experimental validations not only systematically elucidates the anti-inflammatory and antioxidant mechanisms of apigenin but also provides a robust theoretical foundation and experimental evidence supporting this natural flavonoid as a promising therapeutic candidate for ALI.

## Author contributions

Xianglong Kong: conceptualization, investigation, writing – original draft. Yan Liu: project administration, software. Zhigou Zhou: data curation, formal analysis. Liangdong Zhu: investigation, methodology. Xia Ai: project administration, validation. Jiefu Tang: investigation, supervision. Jianjin Guo & Peng Tian & Xia Chen: conceptualization, data curation, supervision, visualization, writing – review and editing.

## Conflicts of interest

The authors declare that there are no competing interests or that could influence the work described in this paper.

## Supplementary Material

RA-OLF-D6RA05523K-s001

RA-OLF-D6RA05523K-s002

## Data Availability

The data supporting the findings of this study are available upon request from the corresponding author. All experimental designs and data collection methods have been meticulously documented to ensure accuracy, transparency, and reproducibility. Supplementary information (SI) is available. See DOI: https://doi.org/10.1039/d6ra05523k.
