# Supplementary material for: Integrated network pharmacology, molecular docking, and experimental validation elucidate the anti-inflammatory and antioxidant mechanisms of apigenin in LPS-induced acute lung injury
Source: RSC Adv. 2026 Jul 23;16(38):42679–96. doi: 10.1039/d6ra05523k (PMC13395130; doi:10.1039/d6ra05523k)
Supplement: Supplementary file 2 [file RA-016-D6RA05523K-s002.pdf]

Supplementary Information (SI) for RSC Advances.
This journal is © The Royal Society of Chemistry 2026

Figure. 2J

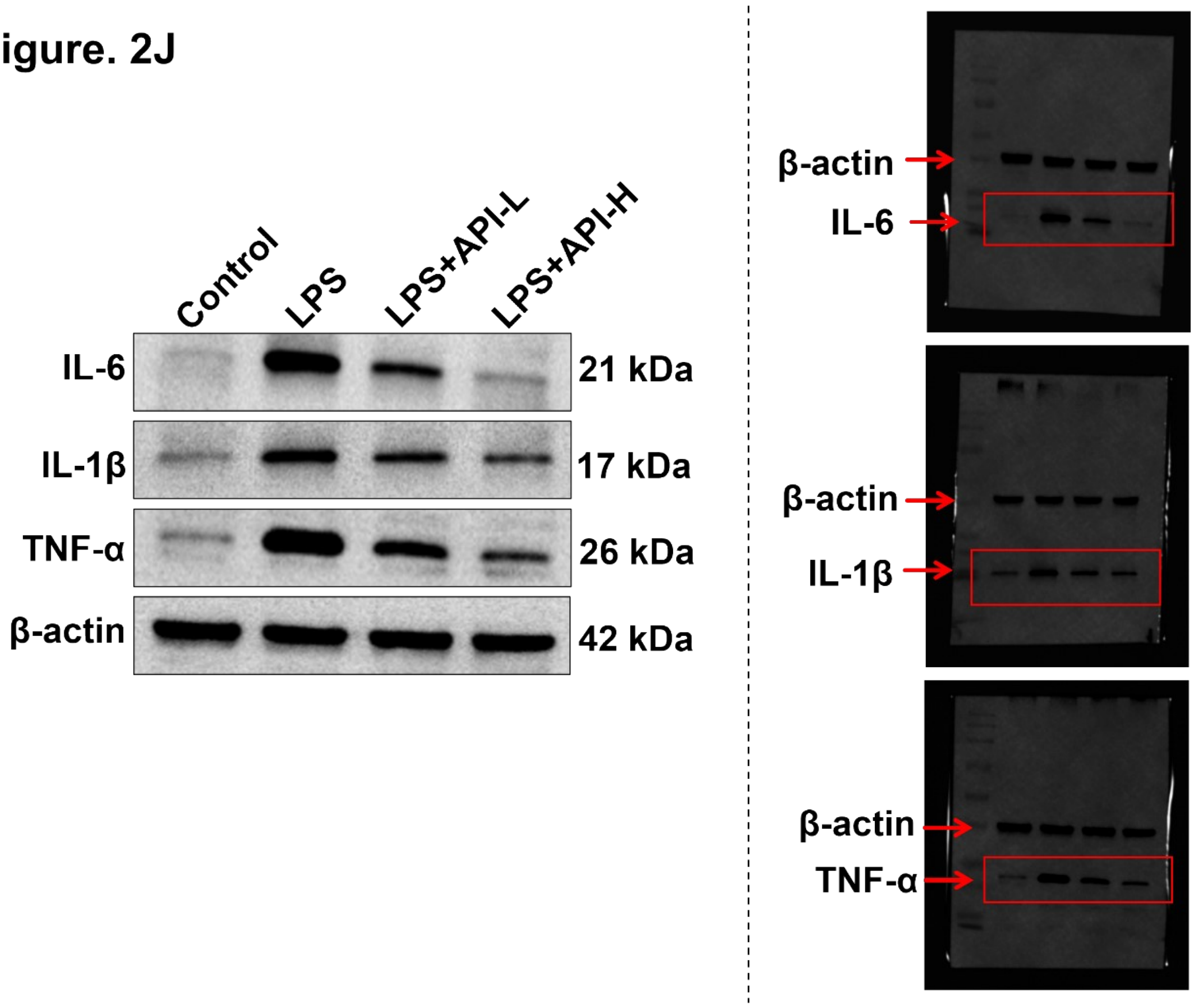

Figure. 6B

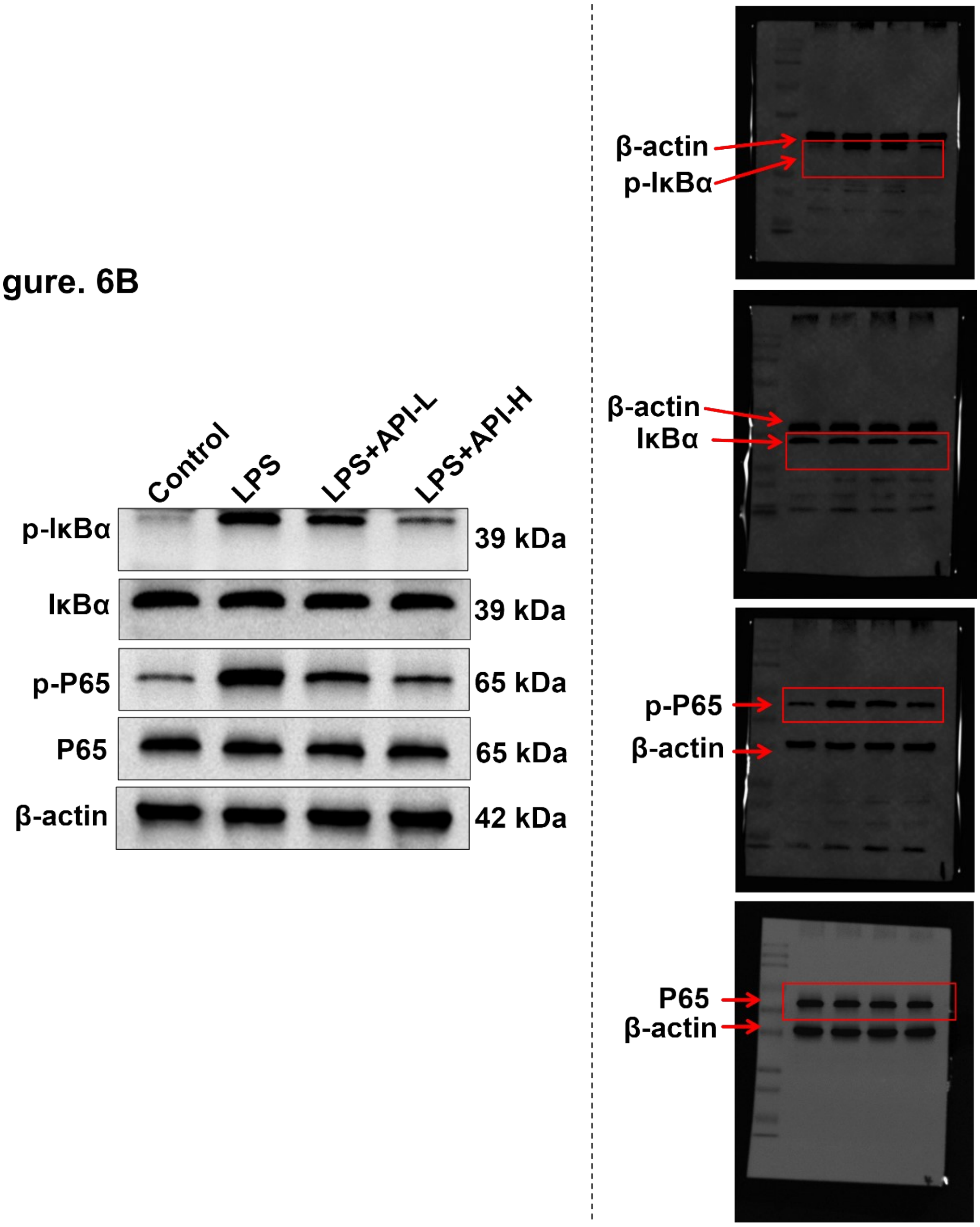

Control
LPS
LPS+API-L
LPS+API-H
p-IκBα
IκBα
p-P65
P65
β-actin
39 kDa
39 kDa
65 kDa
65 kDa
42 kDa
β-actin
p-IκBα
β-actin
IκBα
p-P65
β-actin
P65
β-actin

Figure. 6C

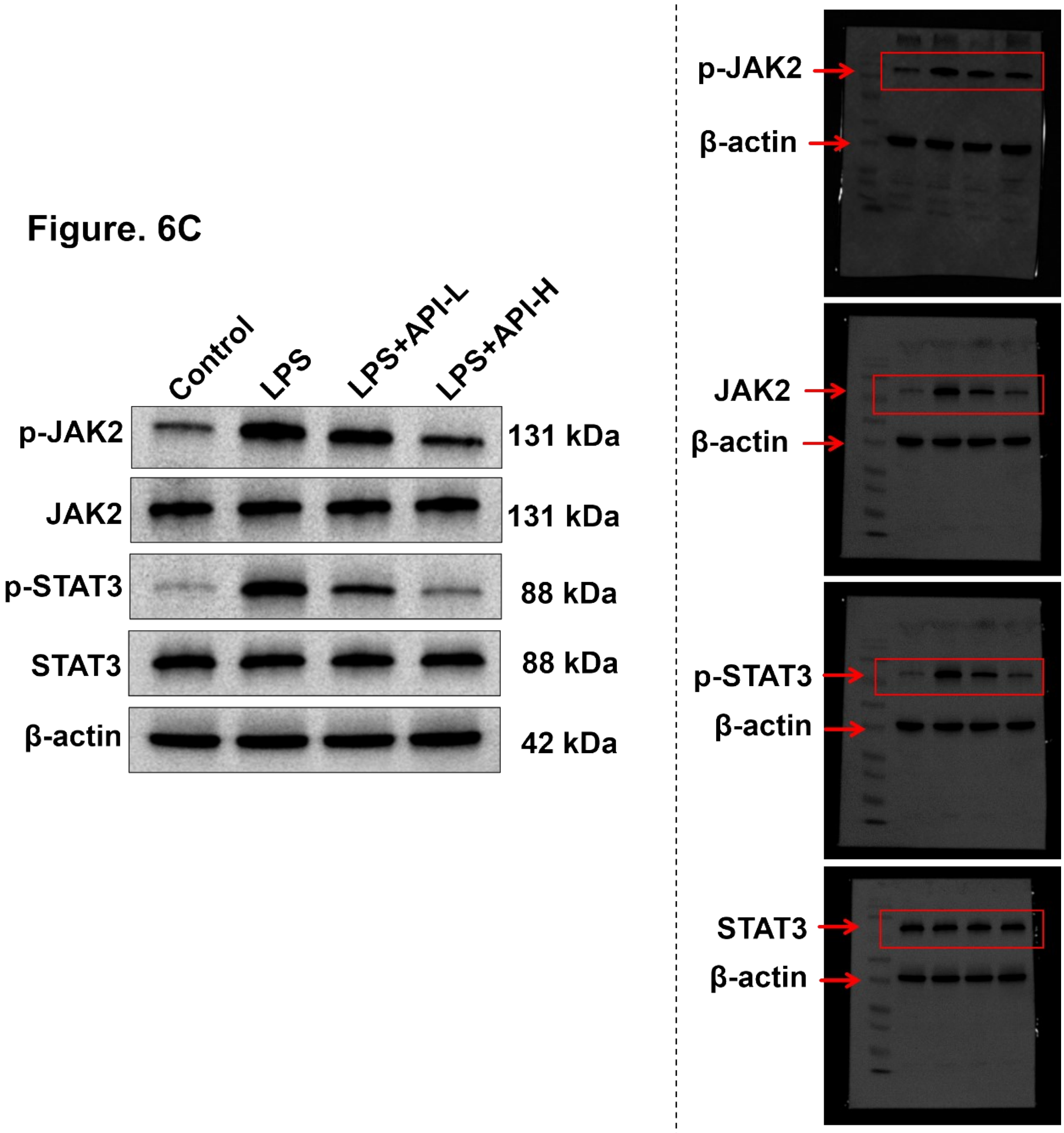

Figure. 7H

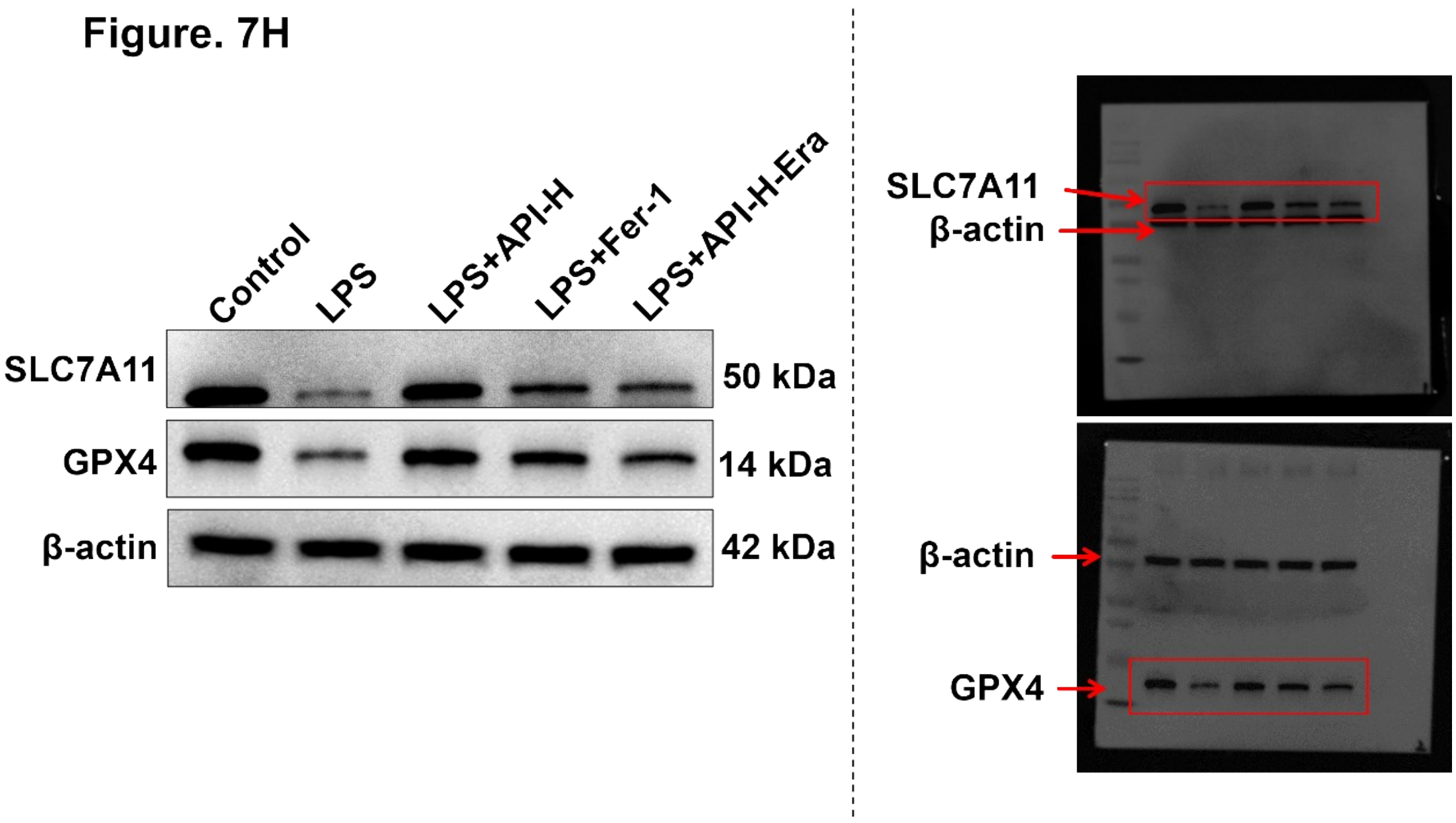

Figure. 9A

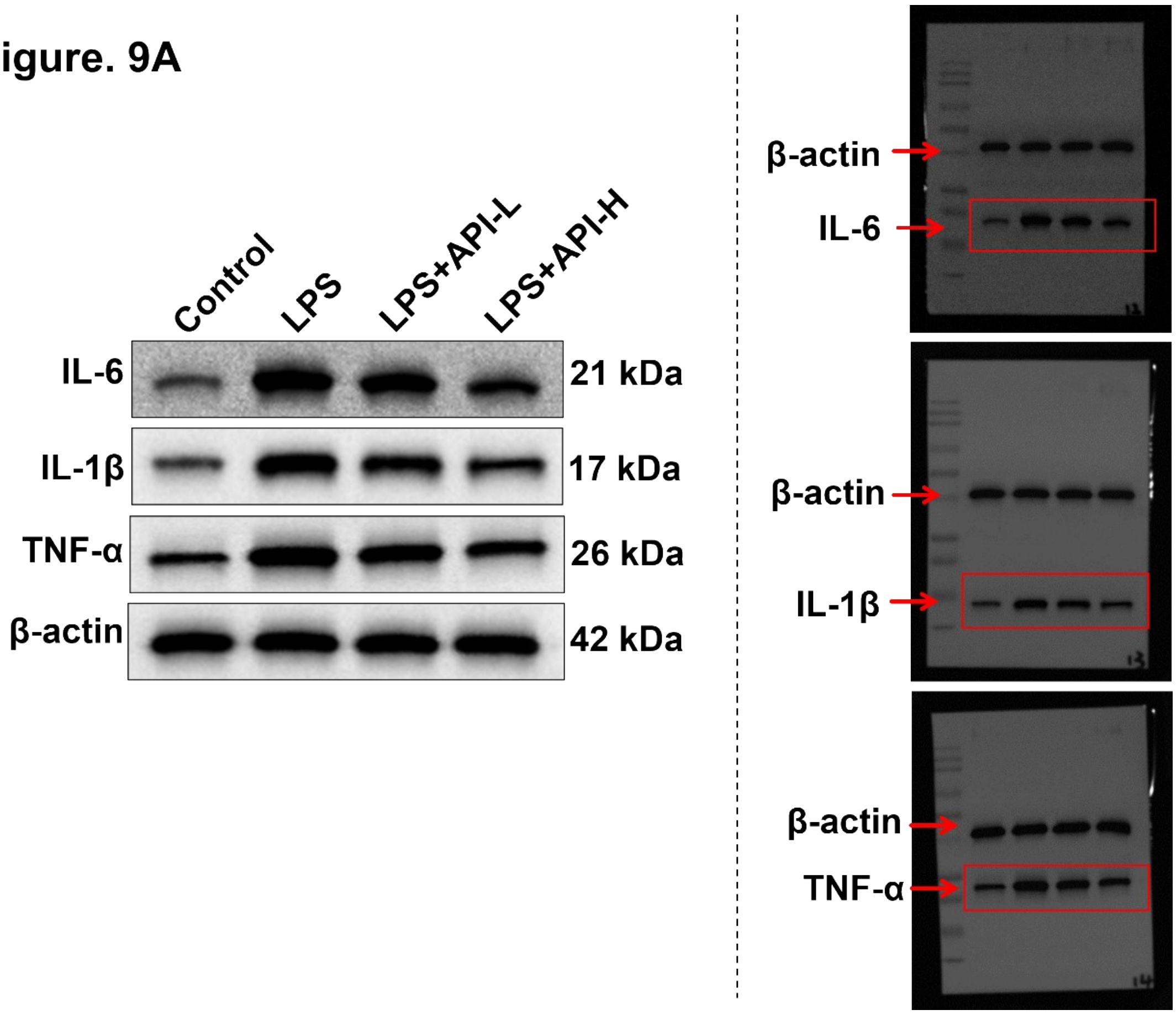

Control
LPS
LPS+API-L
LPS+API-H
IL-6
21 kDa
IL-1β
17 kDa
TNF-α
26 kDa
β-actin
42 kDa
β-actin
IL-6
β-actin
IL-1β
β-actin
TNF-α

Figure. 9B

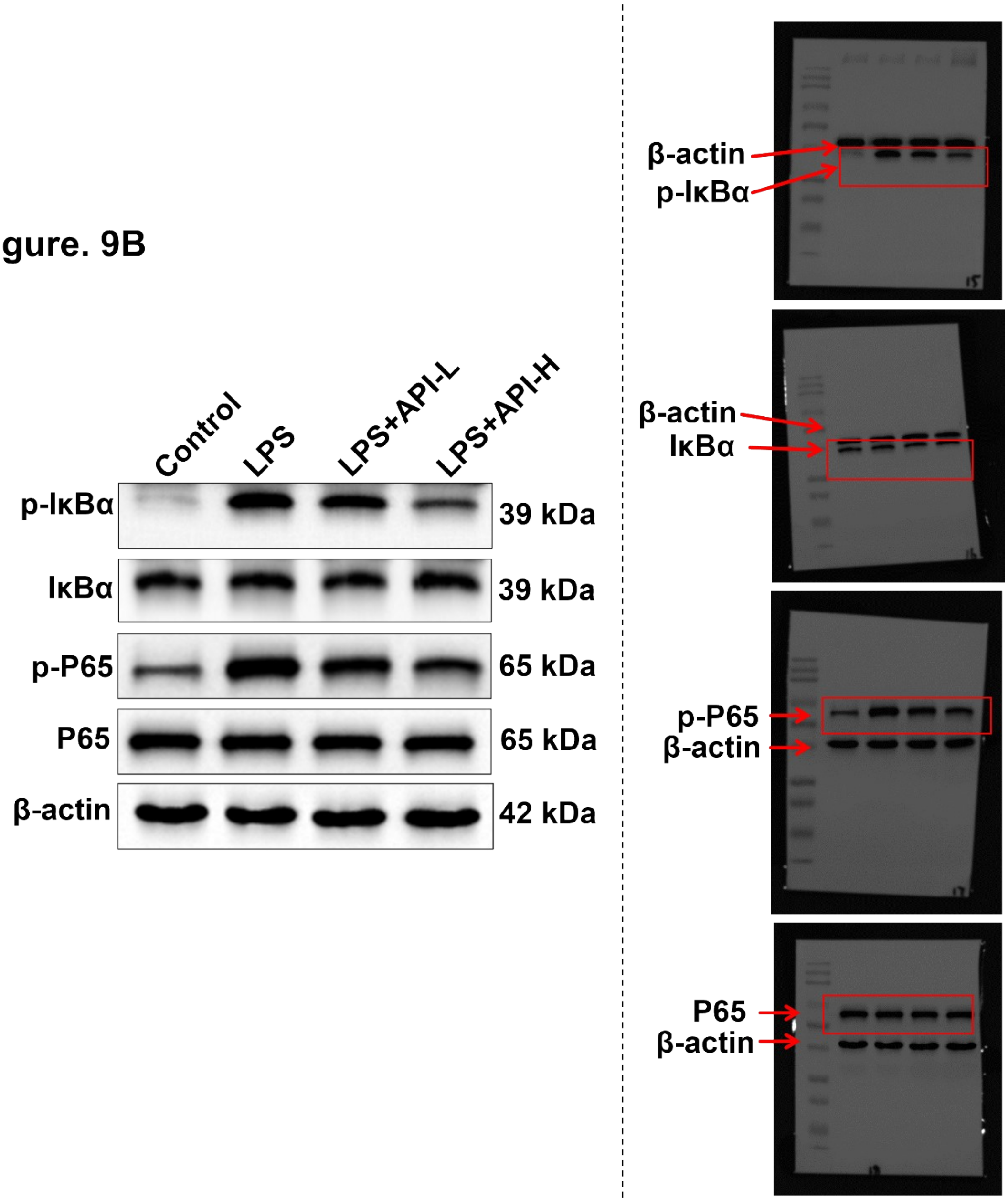
Control
LPS
LPS+API-L
LPS+API-H
p-IκBα
IκBα
p-P65
P65
β-actin
39 kDa
39 kDa
65 kDa
65 kDa
42 kDa
β-actin
p-IκBα
β-actin
IκBα
p-P65
β-actin
P65
β-actin

Figure. 9C

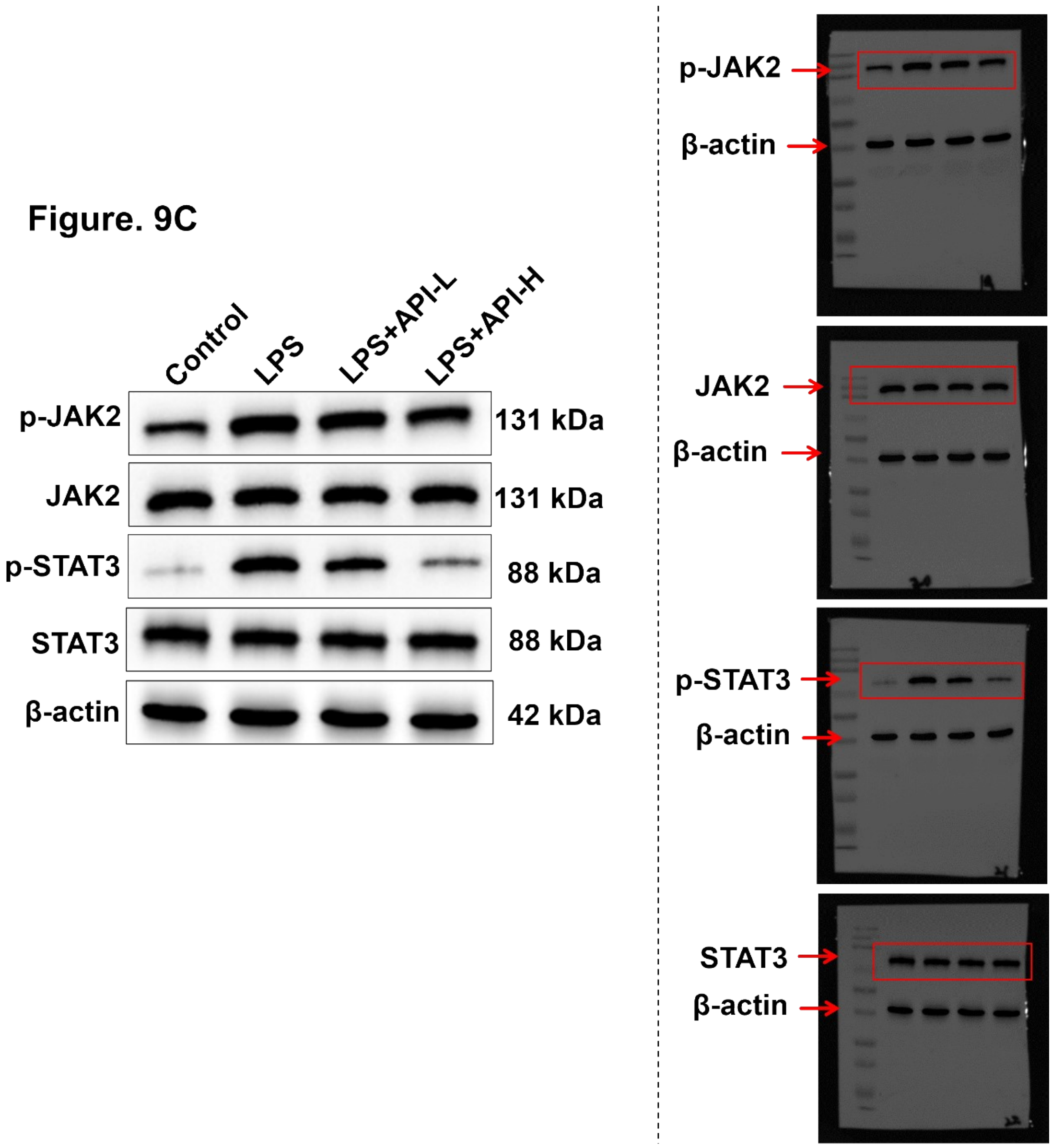

Control
LPS
LPS+API-L
LPS+API-H
p-JAK2
131 kDa
JAK2
131 kDa
p-STAT3
88 kDa
STAT3
88 kDa
β-actin
42 kDa
p-JAK2
β-actin
JAK2
β-actin
p-STAT3
β-actin
STAT3
β-actin

Figure. 10H

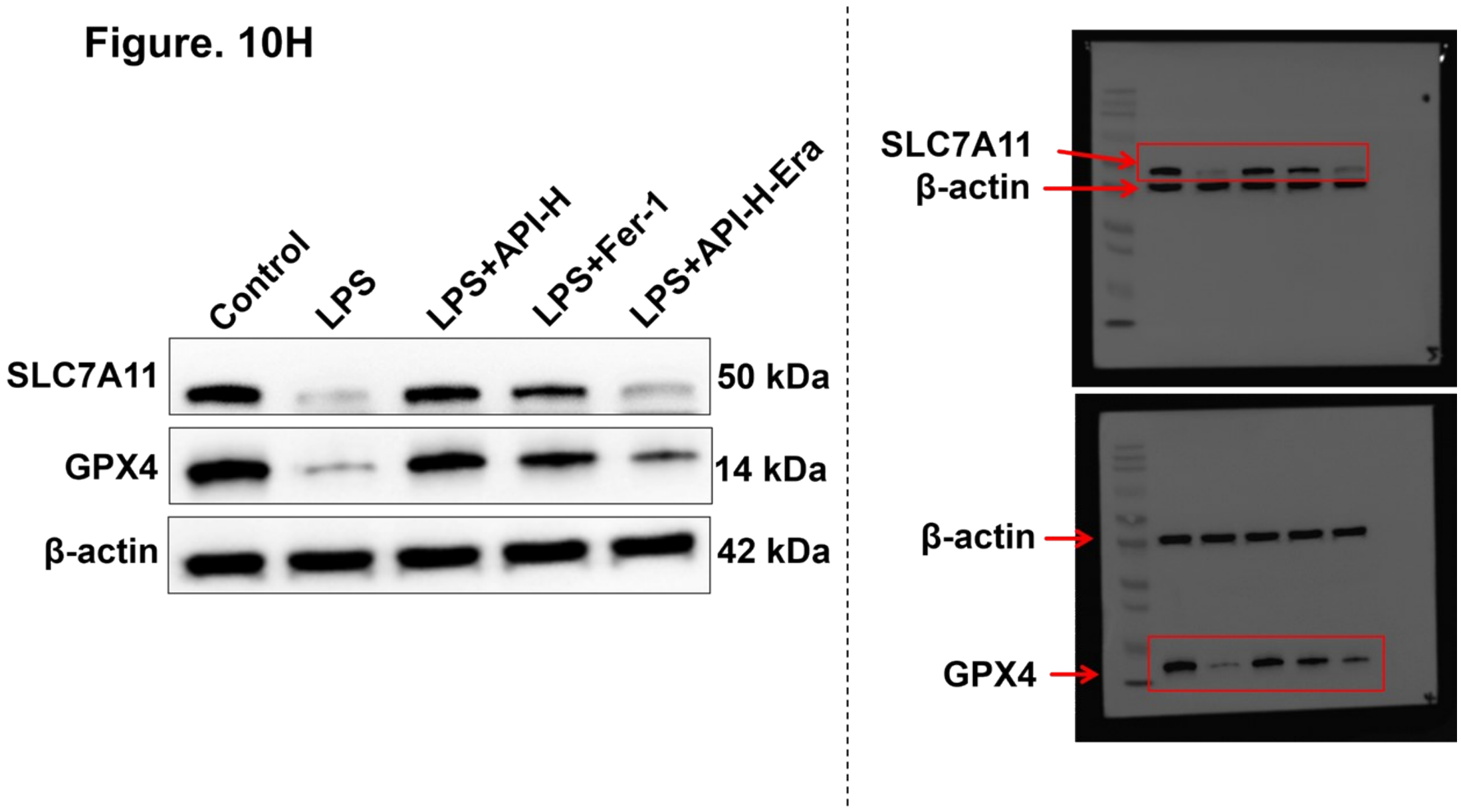
Control
LPS
LPS+API-H
LPS+Fer-1
LPS+API-H-Era
SLC7A11
50 kDa
GPX4
14 kDa
β-actin
42 kDa
SLC7A11
β-actin
β-actin
GPX4
