# Supplementary material for: Integrated network pharmacology, molecular docking, and experimental validation elucidate the anti-inflammatory and antioxidant mechanisms of apigenin in LPS-induced acute lung injury
Source: RSC Adv. 2026 Jul 23. Online ahead of print. doi: 10.1039/d6ra05523k (PMC13395130; doi:10.1039/d6ra05523k)
Supplement: RA-OLF-D6RA05523K-s001 [file RA-OLF-D6RA05523K-s001.pdf]

Supplementary Information (SI) for RSC Advances.
This journal is © The Royal Society of Chemistry 2026

Supplementary Information

Integrated network pharmacology, molecular docking, and experimental validation elucidate the anti-inflammatory and antioxidant mechanisms of apigenin in LPS-induced acute lung injury

Xianglong Kong [a], Yan Liu [a], Zhigou Zhou [a], Liangdong Zhu [b], Xia Ai [b], Jiefu Tang [b], Jianjin Guo [c,*], Peng Tian [b,*], Xia Chen [b,*]

[a] The First Hospital of Changsha, Changsha, 410000, Hunan Province, China

[b] The First Affiliated Hospital of Hunan University of Medicine, Huaihua, 418000, Hunan Province, China

[c] State Key Laboratory of Natural Product Chemistry, College of Chemistry and Chemical Engineering, Lanzhou University, Lanzhou 730000, China

***Corresponding Author**

E-mail address: gjj960326@163.com (Jianjin Guo)

E-mail address: 492880114@qq.com (Peng Tian)

E-mail address: 184895725@qq.com (Xia Chen)

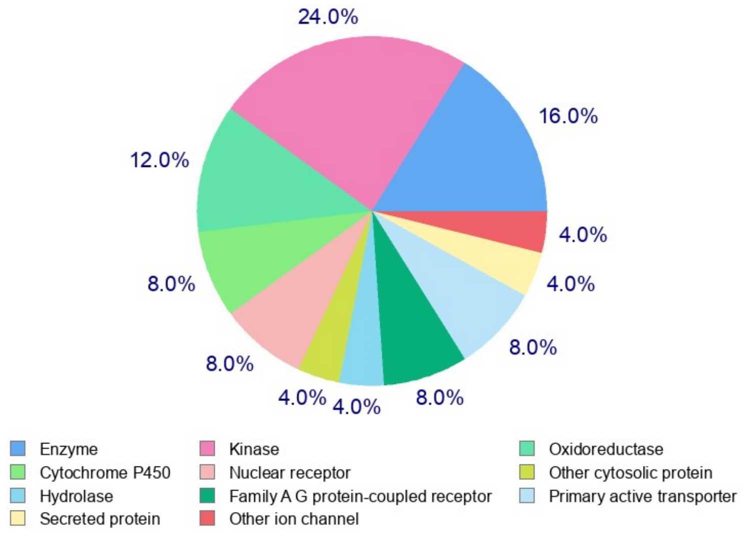

Fig. S1. Distribution prediction of potential targets for Apigenin based on the SwissTargetPrediction database.

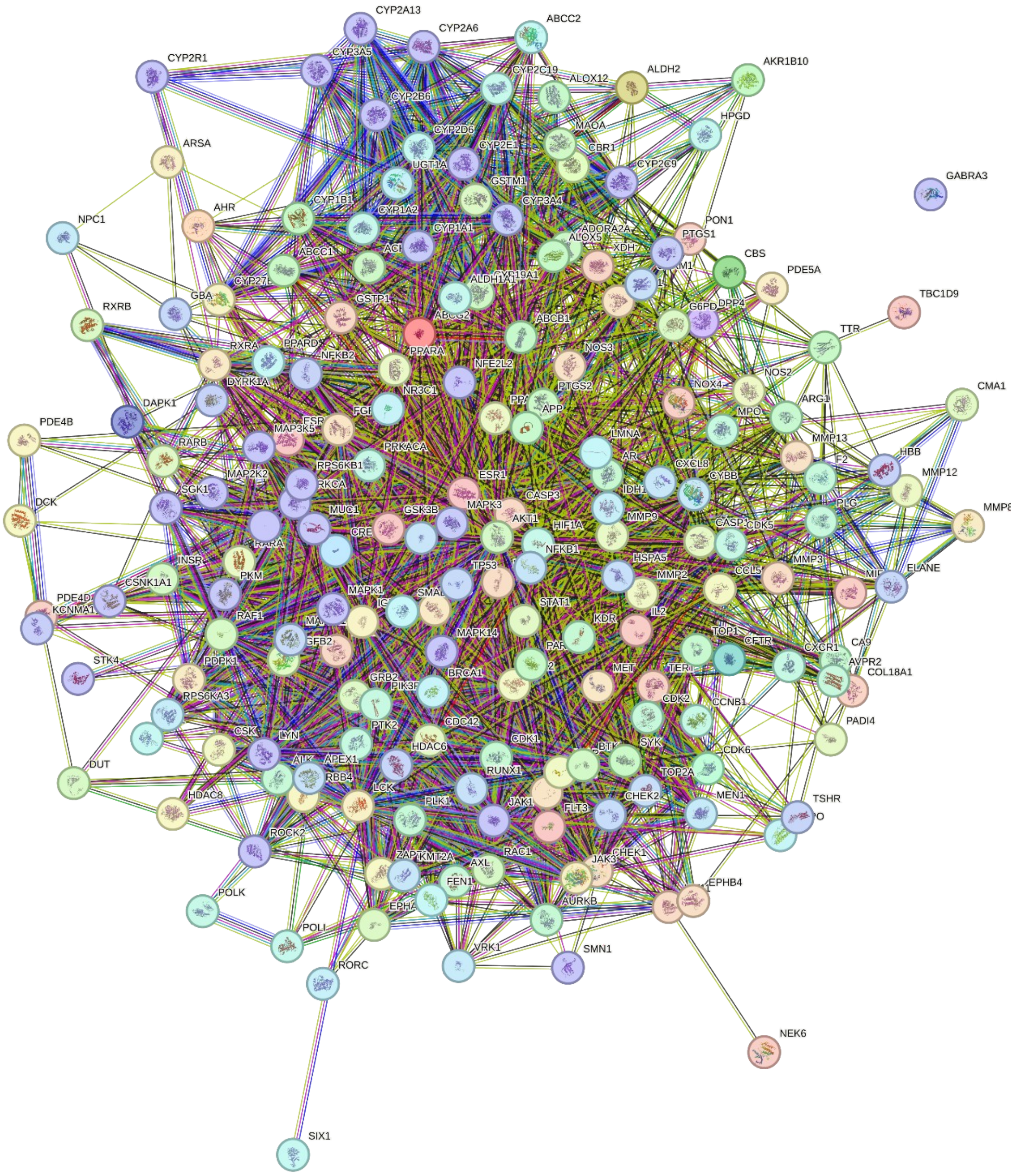

Fig. S2. PPI network of the 161 overlapping targets between Apigenin and lung injury constructed from the STRING database with a confidence score ≥ 0.4.

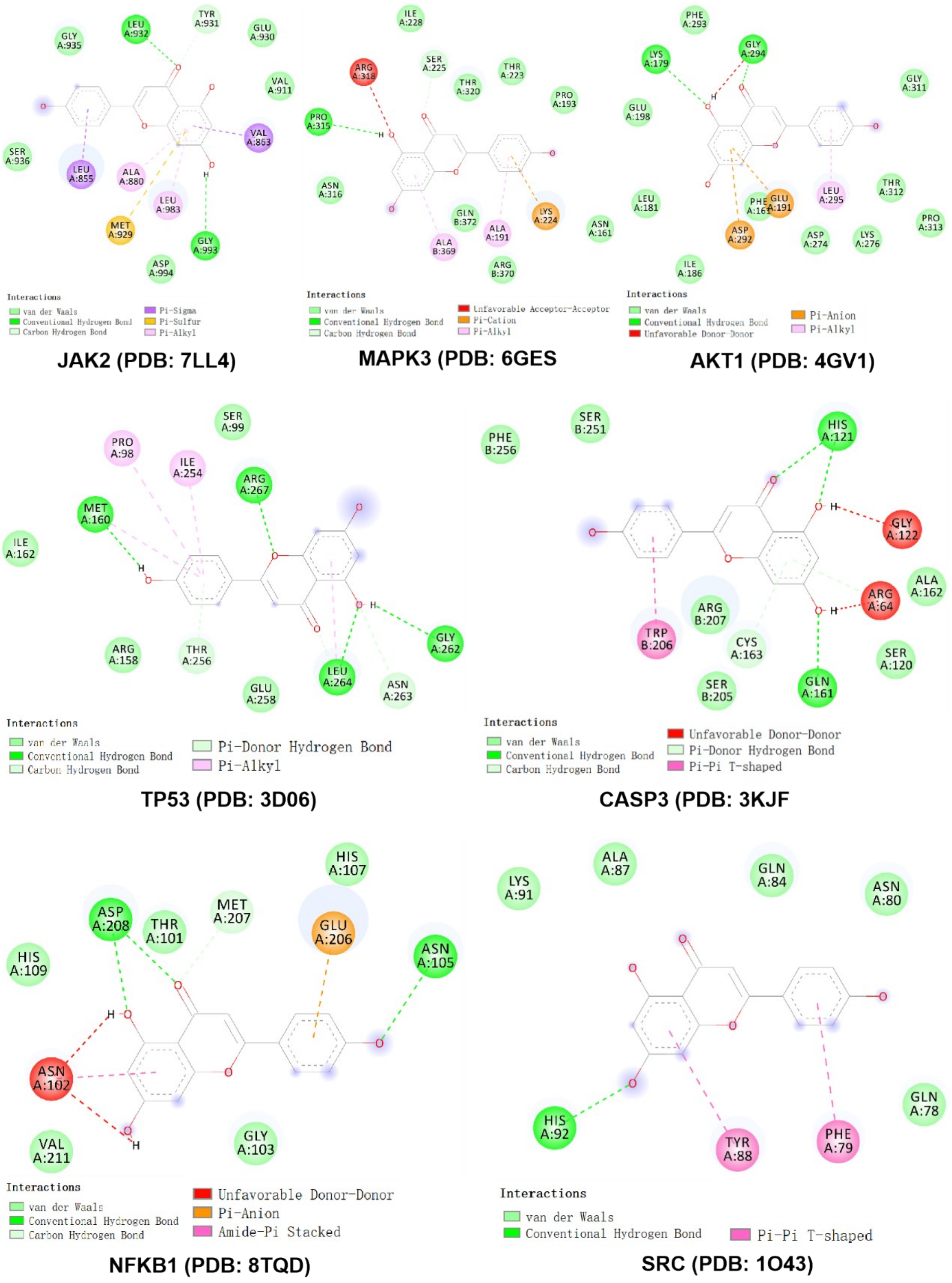

Fig. S3. Two-dimensional visualization of molecular docking interactions between Apigenin and the last seven proteins ranked by binding energy using Discovery Studio.

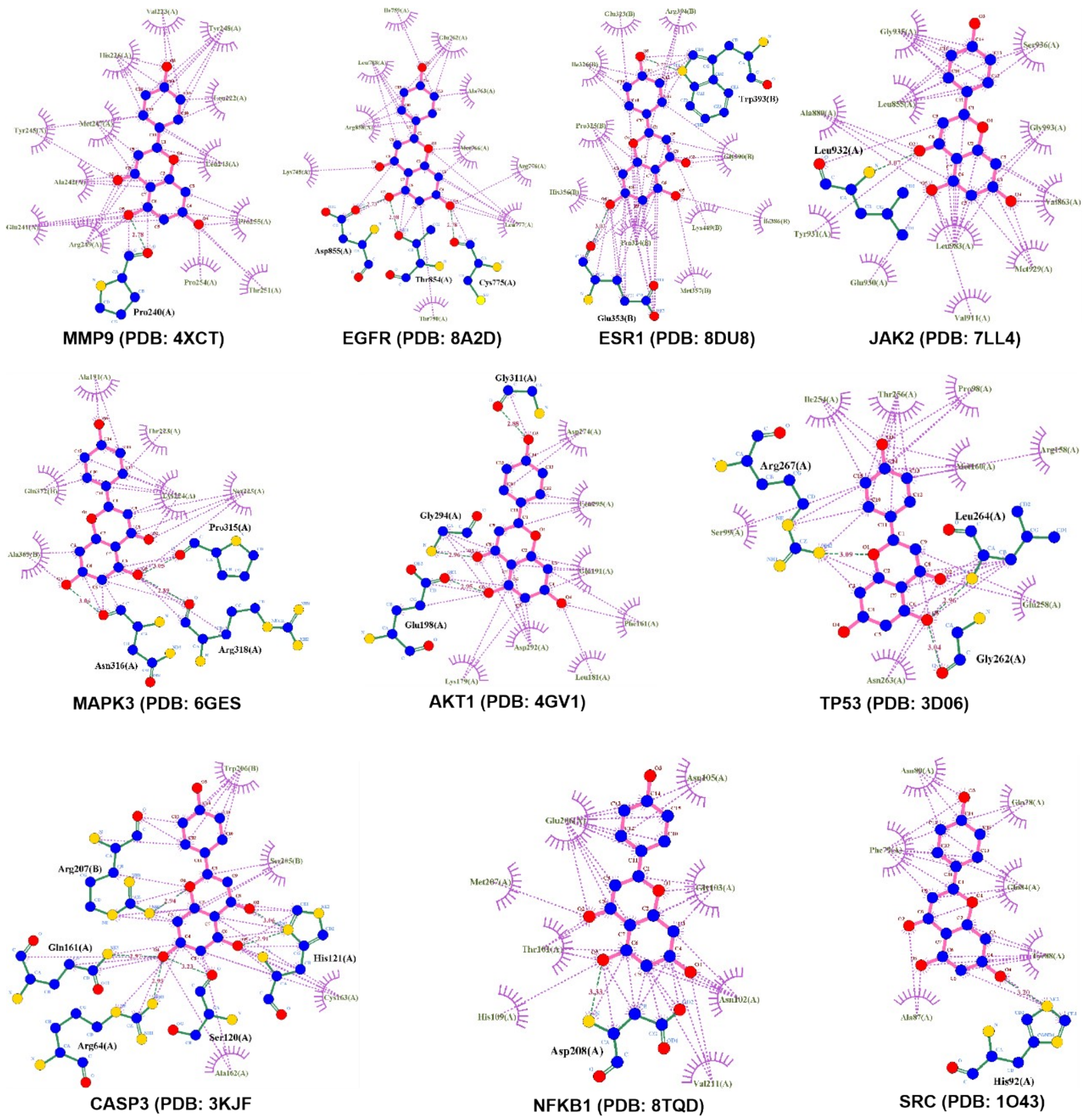

Fig. S4. Two-dimensional visualization of molecular docking interactions between Apigenin and the ten key proteins by binding energy using LigPlus.

Table S1. Primer sequences for RT-qPCR.

Primer name	Forward primer (5'–3')	Reverse primer (5'–3')
IL-6	AGACAGCCACTCACCTCTTCAG	TTCTGCCAGTGCCCTCTTTG
IL-1β	TGGACCTTCCAGGATGAGGACA	GTTCATCTCGGAGCCTGTAGTG
TNF-α	CCTCTCTCTAATCAGCCCTCTG	GAGGACCTGGGAGTAGATGAG
GPX4	CCTCTGCTGCAAGAGCCTCCC	CTTATCCAGGCAGACCATGTGC

SLC7A11	CTTTGTTGCCCTCTCCTGCTTC	CAGAGGAGTGTGCTTGTGGACA
β-actin	CATGTACGTTGCTATCCAGGC	CTCCTTAATGTCACGCACGAT

Table S2. Summary of databases and online tools used in network pharmacology, molecular docking, and molecular dynamics simulations.

Category	Database/Tool	Website	Purpose
Target prediction	PharmMapper	http://www.lilab-ecust.cn/pharmmapper/	Prediction of apigenin potential targets
Target prediction	SwissTargetPrediction	http://www.swisstargetprediction.ch/	Prediction of apigenin potential targets
Target prediction	SEA	http://sea.bkslab.org/	Prediction of apigenin potential targets
Target prediction	ChEMBL	https://www.ebi.ac.uk/chembl/	Retrieval of compound-related targets
Disease target	OMIM	https://omim.org/	Retrieval of lung injury-related targets
Disease target	GeneCards	https://www.genecards.org/	Retrieval of lung injury-related targets
Disease target	TTD	https://db.idrblab.net/	Retrieval of lung injury-related targets
PPI network	STRING	https://cn.string-db.org/	Construction of protein–protein interaction network
Network visualization	Cytoscape	—	Visualization and analysis of PPI network (version 3.10.1)
Enrichment	DAVID	https://david-d.ncifcrf.gov/	GO and KEGG pathway enrichment analysis

analysis			
Protein structure	PDB	https://www.rcsb.org/	Retrieval of target protein crystal structures
Molecular docking	AutoDock Vina	—	Ligand–receptor binding affinity calculation
Molecular dynamics	GROMACS	—	100 ns molecular dynamics simulation (GROMOS 54a7 force field)
