# Supplementary figures and images for: Integrated network pharmacology, molecular docking, and experimental validation elucidate the anti-inflammatory and antioxidant mechanisms of apigenin in LPS-induced acute lung injury
Source: RSC Adv. 2026 Jul 23. Online ahead of print. doi: 10.1039/d6ra05523k (PMC13395130; doi:10.1039/d6ra05523k)

Figure. 2J

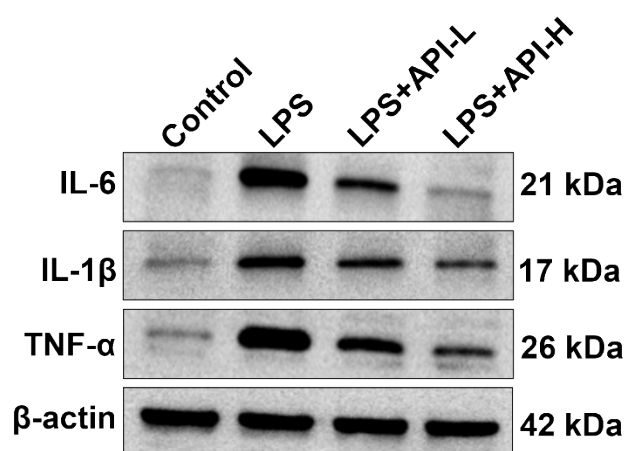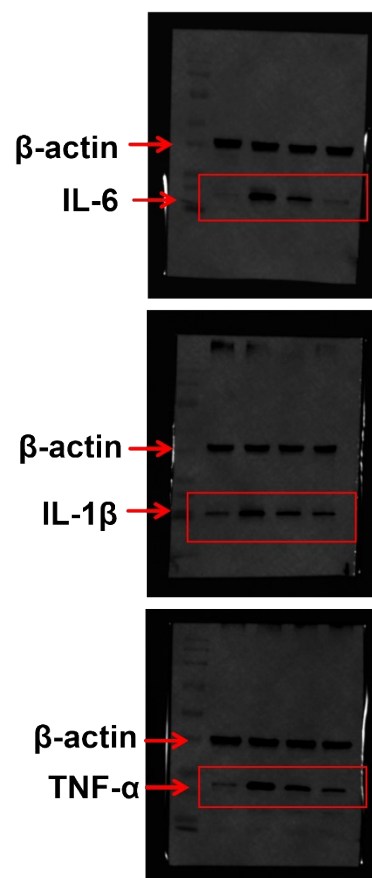

Figure. 6B

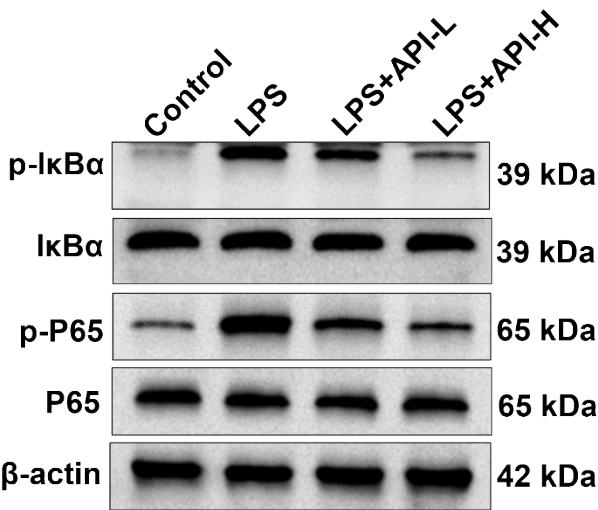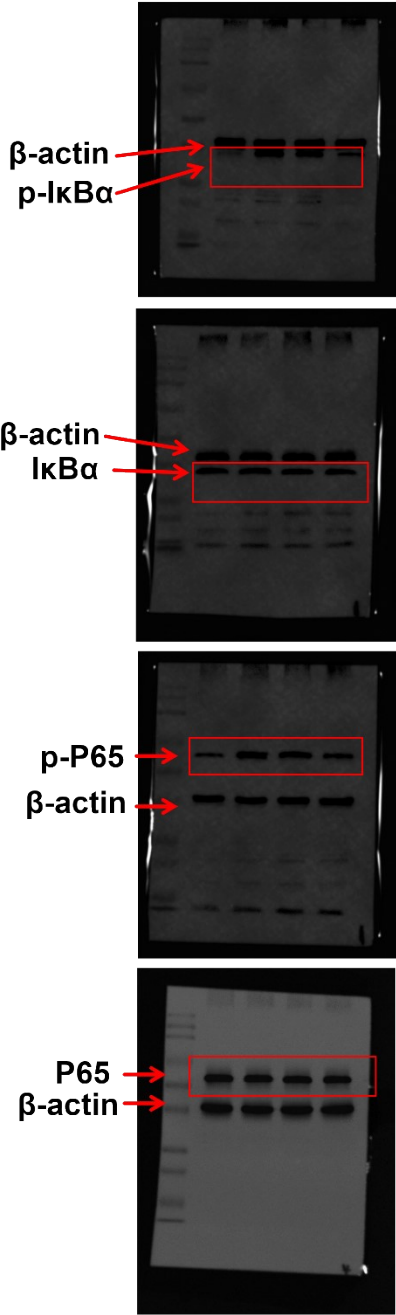

Figure. 6C

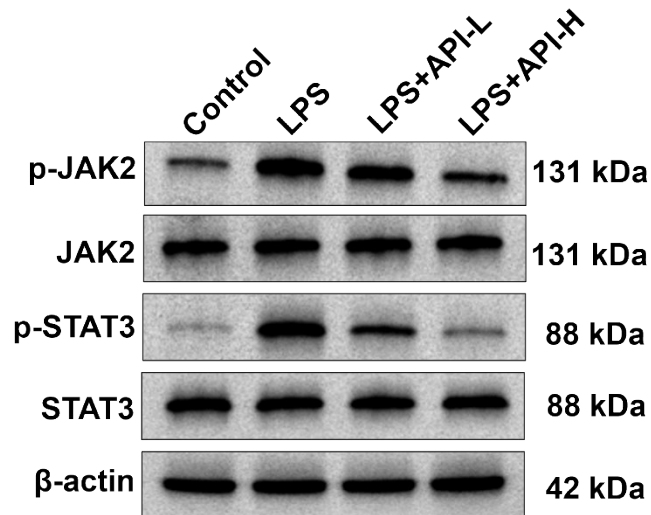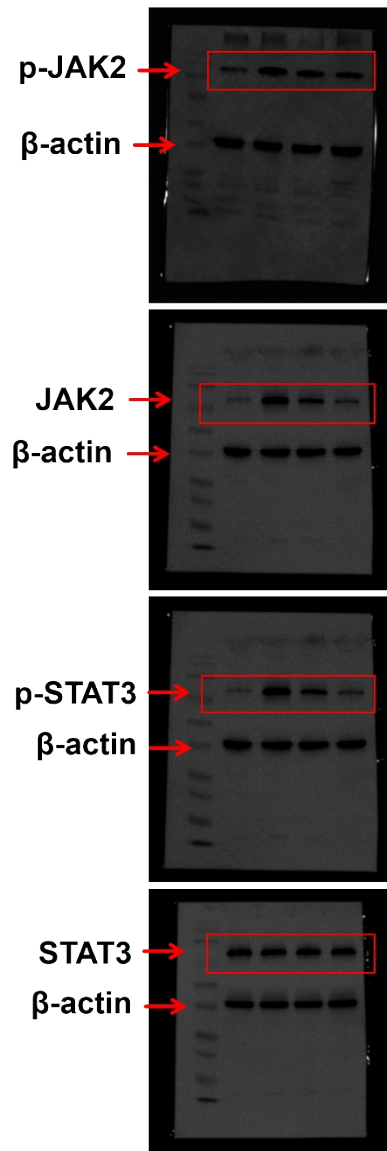

Figure. 7H

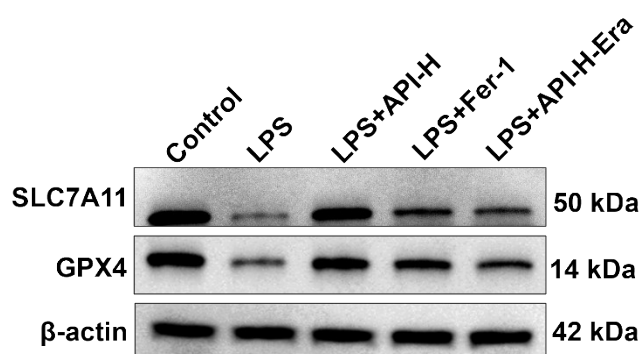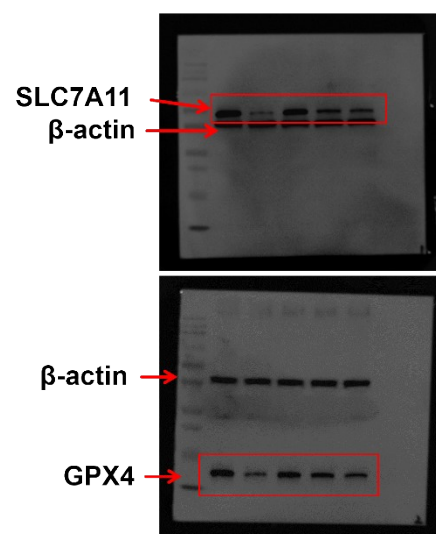

Figure. 9A

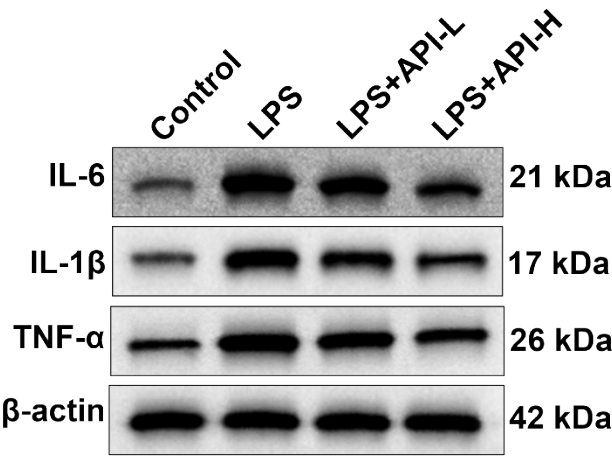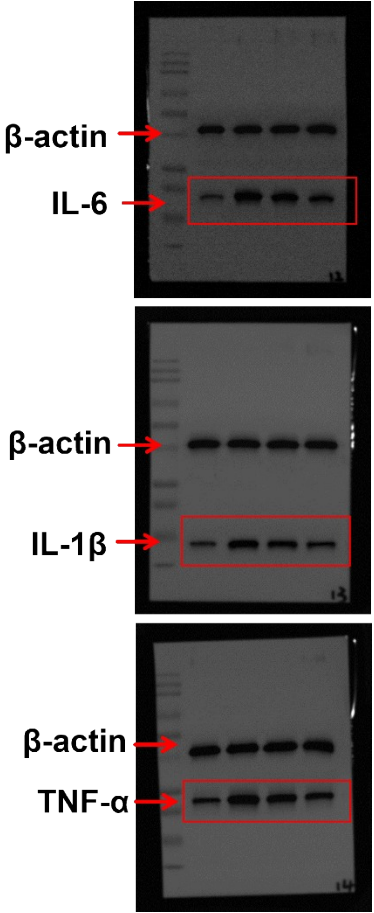

Figure. 9B

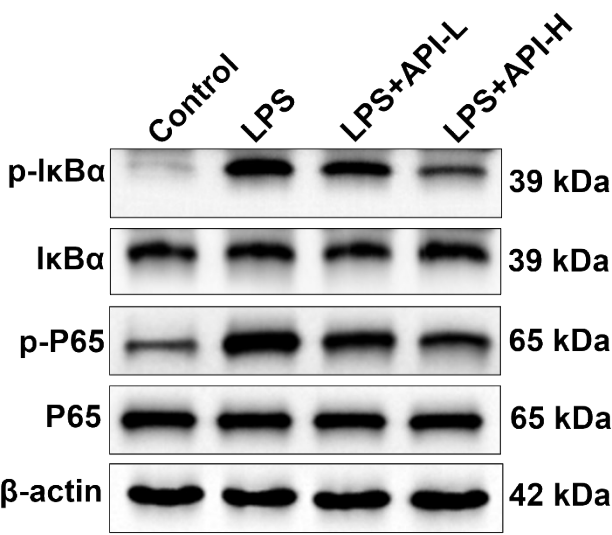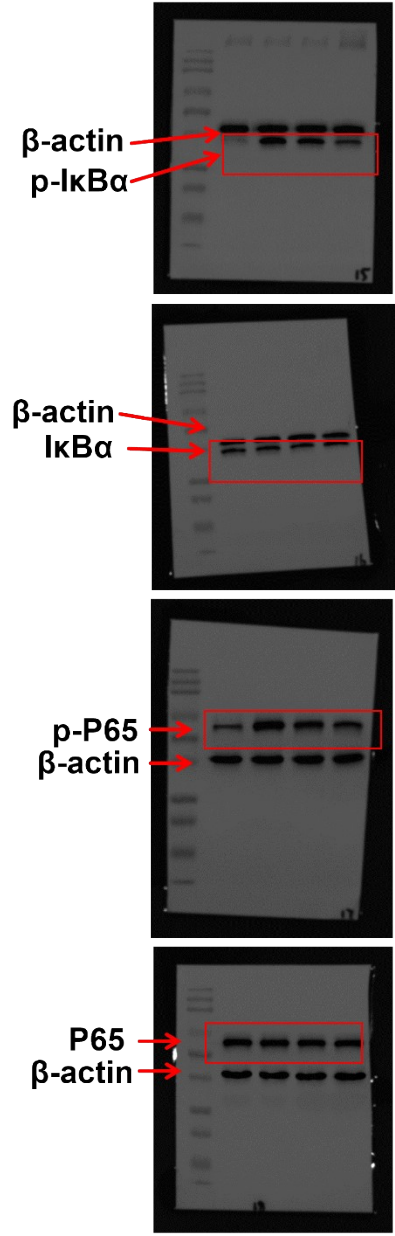

Figure. 9C

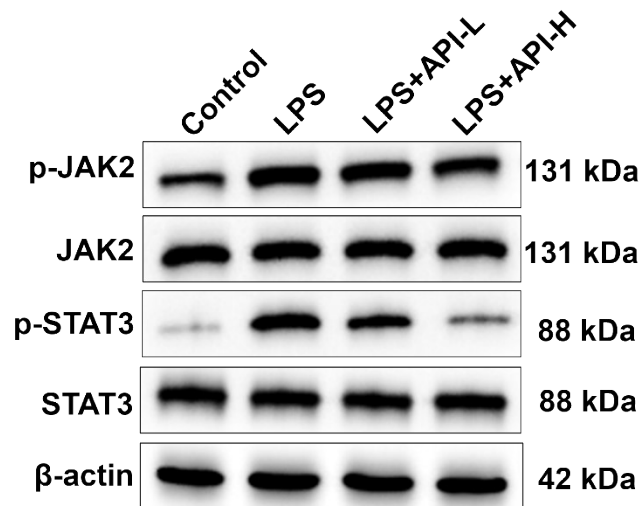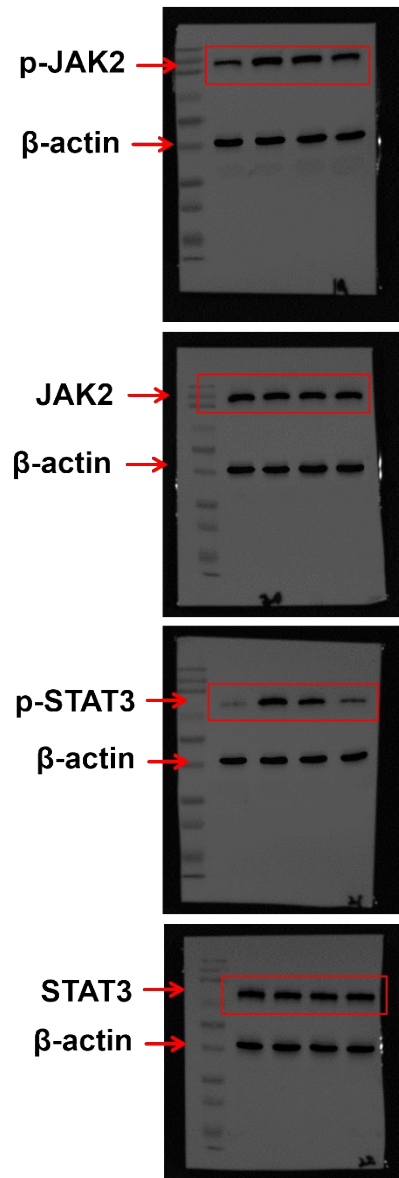

Figure. 10H

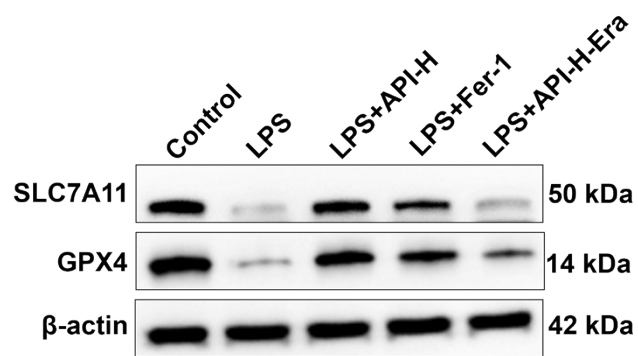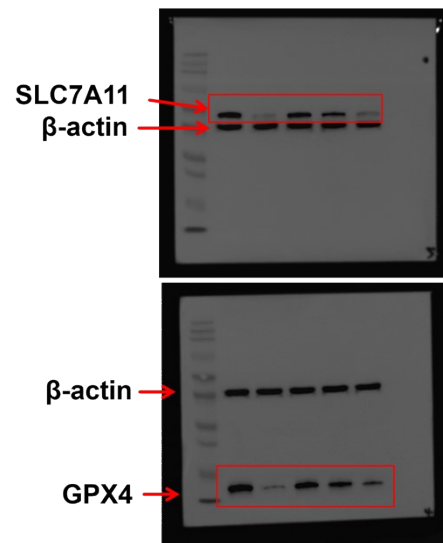

Supplement: RA-OLF-D6RA05523K-s002 [file RA-OLF-D6RA05523K-s002.pdf]
